# Synthesis, Fungicidal Activity and Mode of Action of 4-Phenyl-6-trifluoromethyl-2-aminopyrimidines against *Botrytis cinerea*

**DOI:** 10.3390/molecules21070828

**Published:** 2016-06-24

**Authors:** Chunhui Liu, Zining Cui, Xiaojing Yan, Zhiqiu Qi, Mingshan Ji, Xinghai Li

**Affiliations:** 1 Department of Pesticide Science, Plant Protection College, Shenyang Agricultural University, Shenyang 110866, Liaoning, China; 18809855316@163.com (C.L.); syqizhiqiu@sina.com (Z.Q.); jimingshan@163.com (M.J.); 2 State Key Laboratory for Conservation and Utilization of Subtropical Agro-Bioresources, Integrative Microbiology Research Centre, Guangdong Province Key Laboratory of Microbial Signals and Disease Control, South China Agricultural University, Guangzhou 510642, China; 3 Key Laboratory of Integrated Pest Management in Crops, Ministry of Agriculture, Institute of Plant Protection, Chinese Academy of Agricultural Sciences, Beijing 100193, China; yanxiaojing@caas.cn

**Keywords:** aminopyrimidines, fungicide, structure-activity relationship, mode of action

## Abstract

Anilinopyrimidines are the main chemical agents for management of *Botrytis cinerea*. However, the drug resistance in fungi against this kind of compounds is very serious. To explore new potential fungicides against *B. cinerea*, a series of 4-phenyl-6-trifluoromethyl-2-amino-pyrimidine compounds (compounds **III-1** to **III-22**) were synthesized, and their structures were confirmed by ^1^H-NMR, IR and MS. Most of these compounds possessed excellent fungicidal activity. The compounds **III-3** and **III-13** showed higher fungicidal activity than the positive control pyrimethanil on fructose gelatin agar (FGA), and compound **III-3** on potato dextrose agar (PDA) indicated high activity compared to the positive control cyprodinil. In vivo greenhouse results indicated that the activity of compounds **III-3**, **III-8**, and **III-11** was significantly higher than that of the fungicide pyrimethanil. Scanning electron micrography (SEM) and transmission electron micrography (TEM) were applied to illustrate the mechanism of title compounds against *B. cinerea.* The title compounds, especially those containing a fluorine atom at the *ortho*-position on the benzene ring, could maintain the antifungal activity against *B. cinerea*, but their mechanism of action is different from that of cyprodinil. The present study lays a good foundation for us to find more efficient reagents against *B. cinerea*.

## 1. Introduction

Pyrimidine is an important structural motif to enhance the bioactivity in many agrochemicals, including insecticides, herbicides and fungicides [1,2]. There are many kinds of fungicides containing pyrimidine motifs, such as pyrimethanil, cyprodinil, and mepanipyrim [2,3]. These fungicides are one of the main chemical prevention and control reagents against *B.* *cinerea*, but they are prone to developing fungicidal resistance [3]. Strains of *B.* *cinerea* highly resistant to pyrimethanil have been found in many countries, such as France [4], America [5], Australia [6], Spain [7], Greece [8], Israel [9], and China [10,11]. Leroux et al. have identified three resistant phenotypes towards anilinopyrimidines (e.g., cyprodinil, mepanipyrim and pyrimethanil) since 1999 [4]. Subsequently, Latorre et al. reported that cyprodinil resistant isolates of *B. cinerea* showed cross resistance to the anilinopyrimidines fungicides mepanipyrim and pyrimethanil [12]. Moyano et al. collected resistance to pyrimethanil data in *B.* *cinerea* populations on vegetable crops in Spain and found that three of 42 isolates tested in 1992 (7%) and four of 40 isolates tested in 2000 (10%), were resistant to pyrimethanil [7]. Latorre and Torres reported isolates having simultaneous resistance to anilinopyrimidines, DMIs, phenylpyrroles and hydroxyanilides in *B.* *cinerea* populations from grapevines in Chile [13]. Fungicide resistance is a serious problem that questions the sustainability of the current gray mold control strategy, which relies almost exclusively on fungicides with single-site modes of action [13]. Besides, Yin et al. reported that the resistance factors (RF) of pyrimethanil-resistant isolates from strawberry ranged from 53.0 to 320.1 in Zhejiang Province, China [10]. In addition, the overall resistance frequency of 750 isolates collected in 2012 for cyprodinil was 27% and the frequency of 1060 isolates collected in 2013 was 17% from strawberry fields of seven southern U.S. states [5,14]. Liu et al. also found that in all of the isolates from tomato tested in Henan Province, China, 86% were resistant to pyrimethanil [11]. The data show the development of resistance to anilinopyrimidines in *B.* *cinerea* increases rapidly.

**Figure 1:**
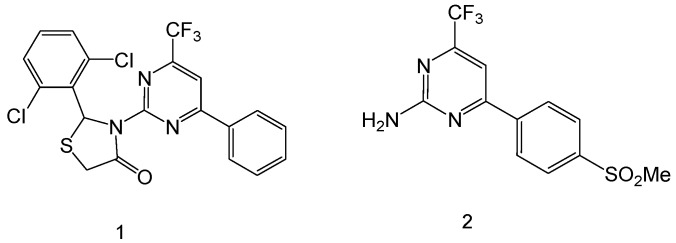
2-amino-4-phenyl-6-trifluoromethyl pyrimidine class of compounds.

**Figure 2:**
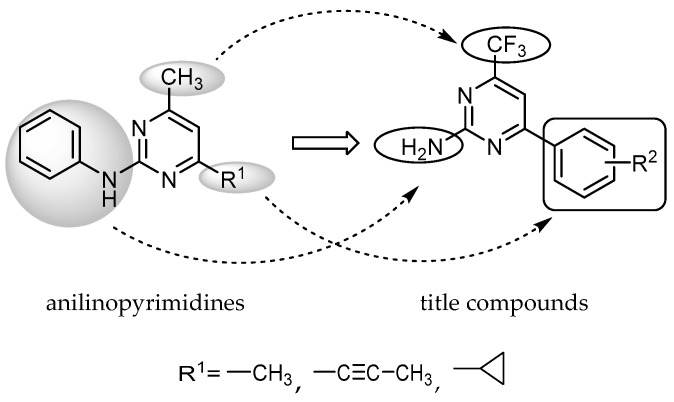
Design strategy for the title compounds.

Toshihiro et al. [15] systematically investigated the structure-activity relationship (SAR) of aminopyrimidines and found that the introduction of aniline to the 2-position, methyl to the 4-position, propargyl, methyl, cyclopropane and other electron-donating groups to the 6-position, respectively, could lead to higher fungicidal activities. 6-Trifluoromethyl pyrimidinamine derivatives were synthesized, and the results showed that they had no fungicidal activity against *B.* *cinerea*. Trifluoromethyl is an active group widely used in drug molecule design [16]. Some 4-trifluoromethyl pyrimidines possessed pharmaceutical bioactivities [17], for example, 2-(2,6-dihalophenyl)-3-pyrimidinyl-1,3-thiazolidin-4-one analogues (Figure 1–1) [18] and (4-(4-(methylsulfonyl) phenyl)-6-(trifluoromethyl)-2-pyrimidinyl) amines (Figure 1–2) [19] belong to the 2-amino-4-phenyl-6-trifluoromethyl pyrimidine class of compounds, and can act as anti-HIV-1 agents and potent and selective cyclooxygenase-2 inhibitors, respectively. There have been no reports however on the agricultural fungicidal activity of 4-substituted phenyl-6-trifluoromethyl-2-aminopyrimidines.

In this paper 4-substituted phenyl-6-trifluoromethyl-2-aminopyrimidines were synthesized to explore new potential fungicides against *B. cinerea*. Studies on the synthetic methodology for preparing 4-phenyl-6-trifluoromethyl-2-aminopyrimidines have been reported. Reports on substituent changes on the benzene ring are scarse, for example, the only modifications on the benzene ring performed to-date involved substitution of the hydrogen atom at the 4-position with methyl [20], fluorine [21,22], methoxy and chlorine [23]. Furthermore, there are no reports on fungicidal activity. The structure and location of the substituent groups on the title compounds underwent various changes, which have a great influence on fungicidal activity of compounds. Compared to pyrimidinamine fungicides, the title compounds possess trifluoromethyl and substituted phenyl groups at position 6 and position 4 of the pyrimidine ring, respectively. The 2-anilino substitutent was transformed into an amino group on the pyrimidine ring (Figure 2). These compounds were then evaluated for their antifungal activities and further study on the mode of action.

## 2. Results and Discussion

### 2.1. Synthesis of 4-Phenyl-6-trifluoromethyl-2-aminopyrimidines

**Scheme 1:**
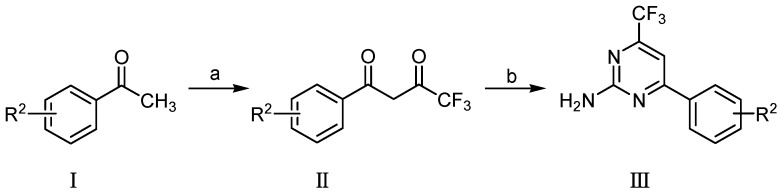
Synthetic route of title compounds. *Reagents and Conditions*: (a) CF_3_COOC_2_H_5_/CH_3_ONa, reflux, 2 h; (b) guanidine hydrochloride/CH_3_ONa, reflux, 6 h;

The syntheses of the title compounds are simple and their yields are good (63%–90%). The synthetic route of the title compounds is shown in Scheme 1.

**Figure 3:**
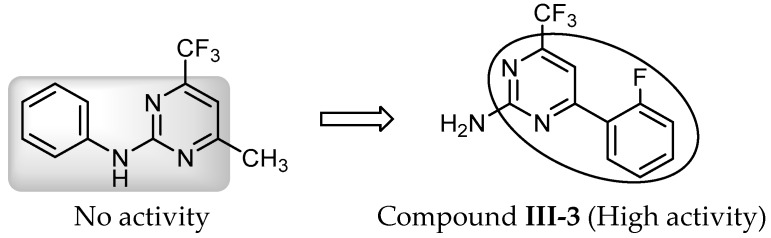
Compared to core framework of anilinopyrimidines and compound **III-3**.

We found the title compounds containing trifluoromethyl group at 6-position, such as compound **III-3**, had very high fungicidal activity. From the structural perspective (Figure 3), the major diference between anilinopyrimidine fungicides and the title compounds relies on their core framework, which is based on pyrimidinamine and on phenylpyrimidine motifs, respectively.

As shown in Table 1, the structure-activity relationships can be summarized in three points. First, for the single substituted benzene structure, whether it is for electron-donating or electron- withdrawing groups, the activity of *ortho*-position is better than that of the *para*-position. Second, the activity of compounds containing halogen is generally higher than that of compounds containing the methyl group and the methoxy group. Third, as far as halogen is concerned, the activity of an *ortho*-position substituent is higher than that of a *para*-position one. Especially for fluorine at the *ortho*-position, compounds showed excellent activity. For example, on FGA, compared with the EC_50_ and EC_80_ values of the positive control pyrimethanil, the relative toxicity of compound **III-3** was 30, 15, respectively, but on PDA, the relative toxicities of compound **III-3** were 13, 46, respectively. In addition, for the benzene ring containing multiple substituents, the activity of *ortho*-position and fluorine atom-containing compounds is still relatively high. The related literature reports that fluorine can be used in medicinal chemistry to modulate the physicochemical properties, such as lipophilicity or basicity. In addition, a fluorine substituent can lead to a change in the preferred molecular conformation and improve the metabolic stability. Due to the major successes of fluorinated compounds in medicinal chemistry, it may be predicted that the number of fluorine containing drugs on the market will continue to increase [24,25,26]. The experimental results proved that the role of the fluorine is consistent with previous reports. Fluorine will also play a continuing role in agricultural chemicals [27].

### 2.2. Fungicidal Activity and Mode of Action of 4-Phenyl-6-trifluoromethyl-2-aminopyrimidines

In this paper, twenty-two compounds were subjected to in vitro and in vivo fungicidal activity screening using the mycelium inoculation method (Table 1 and Table 2). Highly active compound **III-3** was selected, which fungicidal activity was significantly higher than that of pyrimethanil and cyprodinil. Finally, the mode of action of compound **III-3** was studied. With respect to the mode of action of pyrimethanil, cyprodinil and compound **III-3**, they had both similarities and differences. They were similar in both protective and therapeutic effects. Although pyrimethanil and cyprodinil both belong to the pyrimidinamine fungicides, their modes of action are inhibition of methionine biosynthesis [28] and the bioactivity of cytoderm hydrolytic enzymes, respectively [29]; they also had significantly different fungicidal activities in different culture media. Pyrimethanil only exhibits higher fungicidal activity in fructose gelatin agar (FGA) culture medium, but cyprodinil can have higher activity in potato dextrose agar (PDA) culture media. In a screening against five pyrimethanil-resistant strains of *B.* *cinerea*, cyprodinil showed higher activity, but compound **III-3** showed the highest activity. Further studies were performed to understand the mode of action of compound **III-3** and whether cyprodinil and compound **III-3** have different mechanisms of action. The effects of compound **III-3** treatment on mycelial shape and the ultrastructure of *B. cinerea* have both similarities and differences. Scanning electron microscopy (SEM) analysis displayed that the mycelia after compound **III-3** and cyprodinil treatment appeared branched to different degrees. The growth points of mycelia appeared deformed to different degrees, but the growth point deformation after compound **III-3** treatment was more serious than that caused by cyprodinil treatment. Cyprodinil treatment caused serious swelling of mycelia at 3.125 μg/mL, suggesting their mechanisms of action may be different. Transmission electron microscopy (TEM) analysis indicated that the ultrastructure of *B.* *cinerea* changed significantly with cyprodinil and compound **III-3** treatment. Nevertheless, the effect of compound **III-3** on mitochondria is more significant than that of cyprodinil. These results also suggested that the mode of action of compound **III-3** may be different from that of cyprodinil, however, further biochemical evidence, such as their cystathionine lyase and synthase activities, need further research to prove their mechanisms of action are different.

As shown in Table 1, most of the compounds showed high in vitro fungicidal activity on FGA culture medium. Based on EC_50_ and EC_80_ values, compound **III-3** had high activity, whose EC_50_ value was less than 1.0 μg/mL. Compound **III-3** exhibited higher activity than pyrimethanil and cyprodinil. The fungicidal activity of all the compounds was generally low on PDA medium, but the trend was consistent with that on FGA.

As shown in Table 2, twenty-two compounds showed fungicidal activity in vivo against *B. cinerea* at 750 μg/mL. Among them, the activities of compounds **III-3**, **III-8**, **III-10**, **III-11** and **III-13** were higher than that of the positive control pyrimethanil, and were equivalent to that of the fungicide cyprodinil. In general, the in vitro and in vivo activities showed good consistency.

As shown in Table 3, according to the EC_50_ or EC_80_ values and relative toxicity values, compound **III-3** strongly inhibited the mycelia growth compared to pyrimethanil and cyprodinil. The activity of compound **III-3** was the highest against all the pyrimethanil-resistant strains. In addition, some irregularities were caused in colonial growth of *B.* *cinerea* in the presence of compound **III-3**.

**Figure 4:**
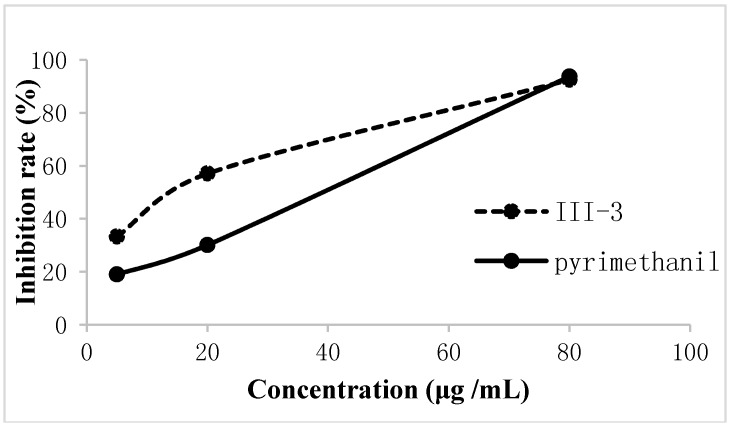
Effect of compound **III-3** on the spore production of *B. cinerea* (FGA).

As shown in Figure 4, compound **III-3** had inhibitory effect on spore production of *B. cinerea*. The inhibition rate reached more than 90%, which was equivalent to pyrimethanil at 80 μg/mL. With decreased concentrations (20 μg/mL and 5 μg/mL), the sporulation inhibition rate of compound **III-3** against *B. cinerea* decreased, while the inhibitory effect was better than that of pyrimethanil.

**Figure 5:**
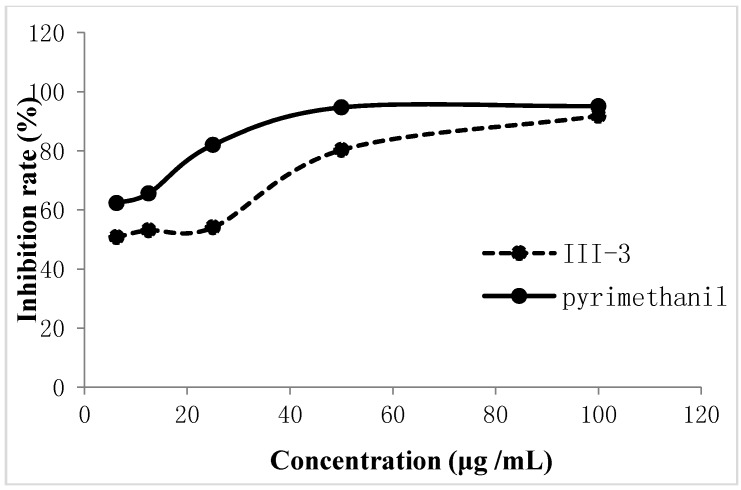
Effect of compound **III-3** on the spore germination of *B cinerea*.

As shown in Figure 5, compound **III-3** had a stronger inhibitory effect on the germination of *B. cinerea* at high concentration, but the overall activity was lower than that of pyrimethanil. At the concentration of 100 μg /mL, the spore germination inhibition rate of compound **III-3** was 91.80%, which was at the same level as the commercial fungicide pyrimethanil. With decreasing concentration, the spore germination inhibition rate of compound **III-3** decreased significantly.

**Figure 6:**
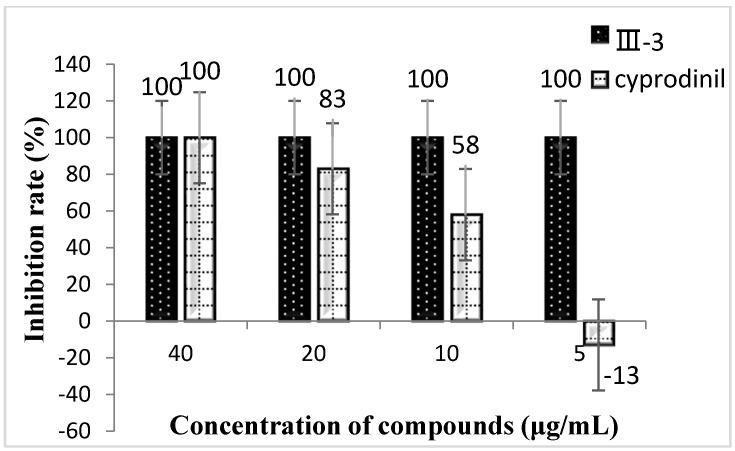
Effect of compound **III-3** on the sclerotia production of *B* *cinerea*.

As shown in Figure 6, compound **III-3** completely inhibited the sclerotia of *B. cinerea.* However, as the concentration decreased, the sclerotia-producing ability of cyprodinil increased. At the concentration of 5 μg /mL, the inhibition rate of cyprodinil is negative, which means it has no inhibition. The result showed that inhibitory effect of compound **III-3** on sclerotia production was better than that of cyprodinil.

**Figure 7:**
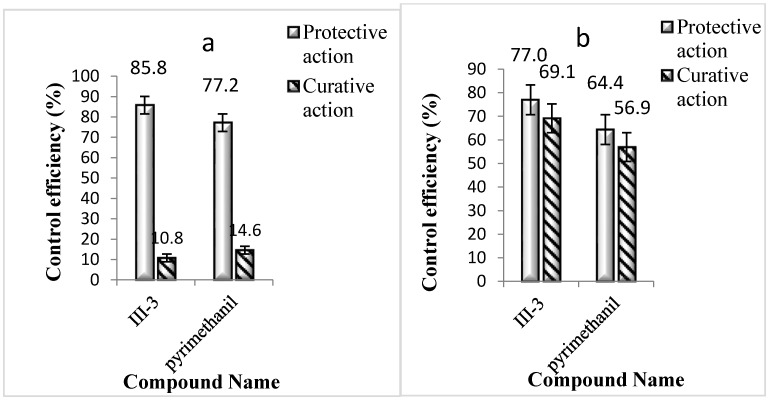
Control efficiency of compound **III-3** against *B. cinerea* (**a**) Control efficiency of compound **III-3** against *B. cinerea* on leaves of cucumber (spore inoculation method**)**; (**b**) control efficiency of compound **III-3** against *B. cinerea* on leaves of tomato (spore inoculation method).

As shown in Figure 7a,b, the results indicate that compound **III-3** had good prevention and treatment effect against *B. cinerea* in cucumber and tomato. Overall, its preventive effect was better than the treatment effect. The activity of compound **III-3** was higher than that of pyrimethanil.

Control efficiency of compound **III-3** against *B. cinerea* on leaves of cucumber was shown in Figure 7a. The plants treated by pyrimethanil displayed obvious water soaked lesions, and the lesions were not only more numnerous but also larger, even with a perforated phenomenon and leaf curl and wilting phenomena were also seen. In addition, the plants treated by compound **III-3** presented edge onset on plant leaves, inconsistent lesion sizes and low incidence in stems and did not appear to show plant wilting and lodging.

Figure 7b shows the control efficiency of compound **III-3** against *B. cinerea* in tomato. Compound **III-3** had excellent prevention and treatment effects, and the activity was higher than that of pyrimethanil at 100 μg/mL.

### 2.3. Scanning Electron Microscopic Analysis

**Figure 8:**
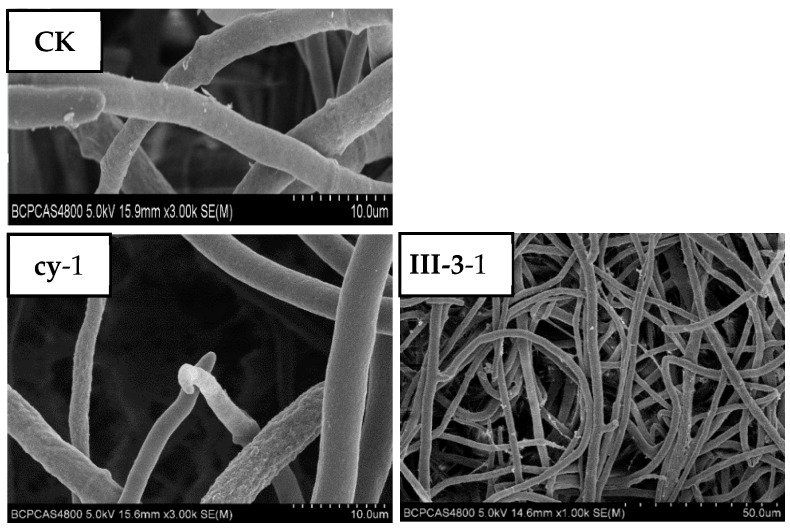

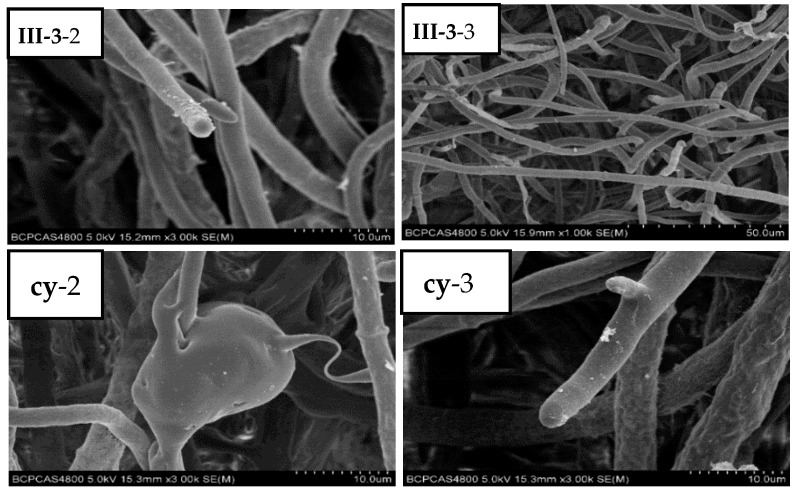
Effect of compound **III-3** treatment on mycelial morphology of *B.* *cinerea*. CK: The blank control; **III-3**-1: Compound **III-3** treatment for 3 days at 0.78 μg/mL; **III-3**-2: Compound **III-3** treatment for 3 days at 3.125 μg/mL; III-3-3: Compound **III-3** treatment for 3 days at 12.5 μg/mL; cy-1: Cyprodinil treatment for 3 days at 0.78 μg/mL; cy-2: Cyprodinil treatment for 3 days at 3.125 μg/mL; cy-3: Cyprodinil treatment for 3 days at 12.5 μg/mL.

The results are shown in Figure 8. The mycelia of blank control were plump and had good extensibility (Figure 8 CK). Nevertheless, the mycelia after compound **III-3** and cyprodinil treatment for 3 days at 0.78 μg/mL are different. The growth point of mycelia display a slight deformity (Figure 8 cy-1). The mycelia with compound **III-3** treatment appear branched (Figure 8 **III-3**-1). As shown in Figure 8 **III-3**-2 and **III-3**-3, after compound **III-3** treatment at 3.125 μg/mL mycelia show serious deformity at the growth points. With the increase of concentration, the number of damaged growth points obviously increased. Unlike compound **III-3** treatment, cyprodinil treatment caused serious swelling of mycelia at 3.125 μg/mL (Figure 8 cy-2), and produced branches at 12.5 μg/mL (Figure 8 cy-3). The results show that the effects of different concentrations on the hyphae are different. The effects of compound **III-3** treatment on the mycelial morphology of *B. cinerea* are manifested in the destruction of the growth points and the increase in the amount of hyphal branching. However, the effects of cyprodinil treatment are manifested in swelling of mycelia and hyphal branching, but not much. The preliminary results thus indicate that they differ in their mode of action.

### 2.4. Transmission Electron Microscopy Analysis

**Figure 9:**
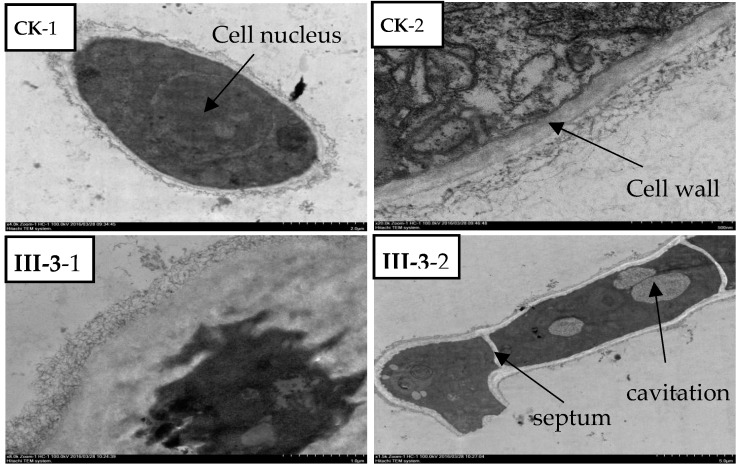

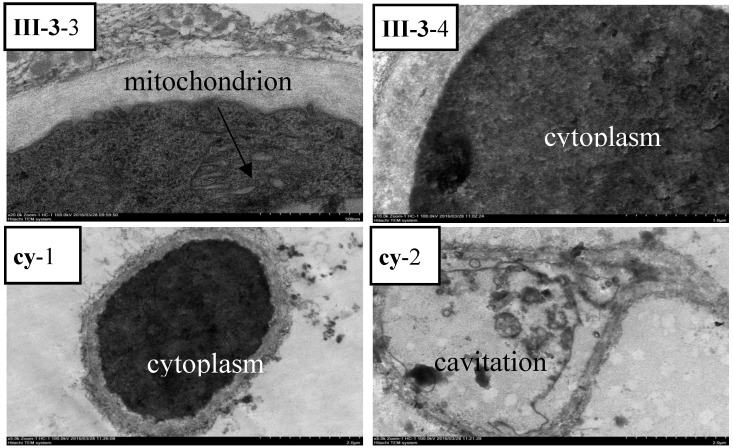
Effect of compound **III-3** treatment on the ultrastructure of *B.* *cinerea.* CK-1: The blank control after 5 days; CK-2: The blank control after 5 days; **III-3**-1: Compound **III-3** treatment for 5 days at 40 μg/mL; **III-3**-2: Compound **III-3** treatment for 5 days at 40 μg/mL; **III-3**-3: Compound **III-3** treatment for 5 days at 20 μg/mL; **III-3**-4: Compound **III-3** treatment for 5 days at 20 μg/mL; cy-1: Cyprodinil treatment for 5 days at 20 μg/mL; cy-2: Cyprodinil treatment for 5 days at 40 μg/mL.

The results are shown in Figure 9. The cell walls of hyphae are regular and smooth without chemical treatment (Figure 9 CK-1, CK-2). Cell nuclear, mitochondria and other organelles are evenly distributed in the cytoplasm (Figure 9 CK-1, CK-2). As shown in Figure 9 CK-2, the cell membrane is clearly visible and intact. When treated with compound **III-3** for 5 days, the cell membrane disappears at 40 μg/mL (Figure 9 **III-3**-1) and mitochondrial matrix appears swollen at 20 μg/mL (Figure 9 **III-3**-3). After compound **III-3** treatment for 5 days at 40 μg/mL, the mycelium shows vacuoles (Figure 9 **III-3**-2). In addition, the cytoplasm is not uniform (Figure 9 **III-3**-4). It is found that compound **III-3** affects the ultrastructure of *B. cinerea*, and causes abnormal alterations such as uneven distribution of the cytoplasm, mitochondrial matrix swelling, membrane structural damage and the formation of vacuoles.

As seen from Figure 9 cy-1, cy-2, the positive control cyprodinil treatment at 40 μg/mL causes serious cavitation. The above results show that effects of compound **III-3** and cyprodinil on the ultrastructure of *B.* *cinerea* are mostly the same. The difference is that compound **III-3** causes mitochondrial matrix swelling, but cyprodinil does not. It is necessary to further study their different action sites.

## 3. Materials and Methods

### 3.1. Instruments

Infrared (IR) spectra were recorded in potassium bromide disks on a Spectrum 65 spectrophotometer (Perkin Elmer, MA, USA). Nuclear magnetic resonance (NMR) spectra were recorded in CDCl_3_ unless indicated otherwise with Bruker 300 MHz or 600 MHz spectrometers (Bruker, NASDAQ, USA), using tetramethylsilane (TMS) as an internal standard. GC-MS: 6890-5973N GC-MS (Agilent, Palo Alto, CA, USA), chromatographic column HP-5MS 5% phenyl methyl siloxane 30 m × 250 μm × 0.25 μm, carrier gas: He; flow rate: 1 mL/min; column temperature, temperature programmed: the initial temperature of 60 °C rose to 200 °C at rate of 20 °C/min, and then rose to 240 °C at rate of 5 °C/min (60 °C–20 °C/min-200 °C–5 °C/min-240 °C); injection port temperature: 230 °C; Shunt model sample: shunt ratio 40:1; assist line temperature: 285 °C. Ion source: 230 °C; quadrupole: 150 °C; ionization mode: EI; acquisition methods: scanning; scan quality range: 35–520 mAU. The solvents and reagents were used as received or were dried prior to use as needed.

### 3.2. Synthetic Procedures

#### 3.2.1. General Synthetic Procedure for 4,4,4-Trifluoro-1-phenylbutane-1,3-dione Compounds **II**

Referring to Scheme 1, to the appropriate acetophenone derivative (0.05 mol) and ethyl trifluoroacetate (0.075 mol) in methanol (20 mL), sodium methoxide solution (0.1 mol of Na + 15 mL of CH_3_OH) was added dropwise at room temperature, and the mixture was refluxed for 2 h. After the methanol was evaporated under vacuum, the residue was dissolved in ethyl acetate (50 mL), washed with 5% HCl (25 mL) and water (25 mL), and dried over sodium sulfate. After the solvent was evaporated under vacuum, the corresponding compound **II** was obtained.

#### 3.2.2. General Procedure for the Preparation of Title Compounds **III**

Compounds **III** were synthesized according to the method given in [16]. To the solution of sodium methoxide (0.09 mol of Na + 10 mL of CH_3_OH), guanidine carbonate (0.08 mol) was added, and the mixture was refluxed for 30 min. The appropriate compound **II** (0.01 mol) was added, then reacted for 6 h. Acetic acid was added dropwise to the solution till pH = 4–5 at 0–5 °C. The reaction solution was filtered, and the filter cake was washed with water (15 mL × 2). The crude product was recrystallized from methanol to give pure compound **III-1** to **III-22**. The nuclear magnetic resonance (NMR), infrared (IR), and mass spectrum (MS) data were as follows:

*4-Phenyl-6-trifluoromethyl-2-aminopyrimidine* (**III-1**): White crystals, yield 89%, m.p. 130–131 °C (130–132 °C) (Rawal et al. [18], 2007). ^1^H-NMR (CDCl_3_, 300 MHz), δ (ppm): 5.47 (br, 2H, NH_2_), 7.35 (s, 1H, py-H), 7.47–8.06 (m, 5H, C_6_H_5_). IR (KBr), ν (cm^−1^): 3323, 3208, 1640, 1600. MS (EI), *m*/*z*: 239 (M^+^, 100%).

*4-(2-Methylphenyl)-6-trifluoromethyl-2-aminopyrimidine* (**III-2**): White crystals, yield 76%, m.p. 135–136 °C ^1^H-NMR (CDCl_3_, 300 MHz), δ (ppm): 2.53 (s, 3H, CH_3_), 5.45 (br, 2H, NH_2_), 6.29 (s, 1H, py-H), 7.25–7.56 (m, 4H, C_6_H_4_). IR (KBr), ν (cm^−1^): 3310, 3163, 1677, 1633. MS (EI), *m*/*z*: 253 (M^+^, 82%).

*4-(2-Fluorophenyl)-6-trifluoromethyl-2-aminopyrimidine* (**III-3**): White crystals, yield 83%, m.p. 116–117 °C, ^1^H-NMR (CDCl_3_, 600 MHz), δ (ppm): 5.45 (br, 2H, NH_2_), 7.18–8.10 (m, 5H, C_6_H_4_ + py-H); IR (KBr), ν (cm^−1^): 3324, 3210, 1634, 1585. MS (EI), *m*/*z*: 257 (M^+^, 100%).

*4-(2-Chlorophenyl)-6-trifluoromethyl-2-aminopyrimidine* (**III-4**): White crystals, yield 79%, m.p. 118–120 °C, ^1^H-NMR (CDCl_3_, 600 MHz), δ (ppm): 5.57 (s, 2H, NH_2_), 7.27–7.74 (m, 5H, C_6_H_4_ + py-H); IR (KBr), ν (cm^−1^): 3326, 3210, 1635, 1584. MS (EI), *m*/*z*: 272.9 (M^+^, 100%).

*4-(2-Bromophenyl)-6-trifluoromethyl-2-aminopyrimidine* (**III-5**): White crystals, yield 77%, m.p. 138–140 °C, ^1^H-NMR (CDCl_3_, 600 MHz), δ (ppm): 5.14 (s, 2H, NH_2_), 7.25–7.71 (m, 5H, C_6_H_4_ + py-H); IR (KBr), ν (cm^−1^): 3325, 3212, 1634, 1555. MS (EI), *m*/*z*: 316.9 (M^+^, 100%).

*4-(2-Trifluoromethylphenyl)-6-trifluoromethyl-2-aminopyrimidine* (**III-6**): White crystals, yield 76%, m.p. 100–101 °C, ^1^H-NMR (CDCl_3_, 600 MHz), δ (ppm): 5.40 (s, 2H, NH_2_), 7.25–7.89 (m, 5H, C_6_H_4_ + py-H); IR (KBr), ν (cm^−1^): 3505, 3331, 1633, 1472. MS (EI), *m*/*z*: 306.9 (M^+^, 100%).

*4-(2-Methoxyphenyl)-6-trifluoromethyl-2-aminopyrimidine* (**III-7**): Light yellow crystals, yield 75%, m.p. 95–97 °C, ^1^H-NMR (CDCl_3_, 600 MHz), δ (ppm): 5.43–5.45 (d, 2H, NH_2_), 7.02–7.89 (m, 5H, C_6_H_4_ + py-H), 3.91 (s, 3H, OCH_3_); IR (KBr), ν (cm^−1^): 3334, 3202, 1638, 1584. MS (EI), *m*/*z*: 268.9 (M^+^, 100%).

*4-(2-Hydroxyphenyl)-6-trifluoromethyl-2-aminopyrimidine* (**III-8**): Light yellow solid, yield 80%, m.p. 180–185 °C, ^1^H-NMR (CDCl_3_, 600 MHz), δ (ppm): 5.29(s, 2H, NH_2_), 6.96–7.81(m, 5H, C_6_H_4_ + py-H), 13.05 (s,1H, OH); IR (KBr), ν (cm^−1^): 3318, 3189, 1646, 1591. MS (EI), *m*/*z*: 255 (M^+^, 100%).

*4-(4-Methoxyphenyl)-6-trifluoromethyl-2-aminopyrimidine* (**III-9**): White crystals, yield 82%, m.p. 193–194 °C (192–193 °C) (Bonacorso et al. [23], 2008). ^1^H-NMR (CDCl_3_, 300 MHz), δ (ppm): 3.88 (s, 3H, CH_3_), 5.40 (br, 2H, NH_2_), 7.00 (d, 2H, *J* = 8.81 Hz, C_6_H_2_), 7.28 (s, 1H, py-H), 8.03 (d, 2H, *J* = 8.81 Hz, C_6_H_2_). IR (KBr), *ν* (cm^−1^): 3333, 3219, 1632, 1596. MS (EI), *m*/*z*: 269 (M^+^, 100%).

*4-(4-Methylphenyl)-6-trifluoromethyl-2-aminopyrimidine* (**III-10**): White crystals, yield 87%, m.p. 180–181 °C. ^1^H-NMR (CDCl_3_, 300 MHz), δ (ppm): 2.43 (S, 3H, CH_3_), 5.37 (br, 2H, NH_2_), 7.30 (d, 2H, *J* = 8.10 Hz, C_6_H_2_), 7.32 (s, 1H, py-H), 7.94 (d, 2H, *J* = 8.10 Hz, C_6_H_2_). IR (KBr), ν (cm^−1^): 3313, 3178, 1640, 1596. MS (EI), *m*/*z*: 253 (M^+^, 100%).

*4-(4-Ethylphenyl)-6-trifluoromethyl-2-aminopyrimidine* (**III-11**): Yellow crystals, yield 90%, m.p. 152–153 °C. ^1^H-NMR (CDCl_3_, 300 MHz), δ (ppm): 1.27 (t, 3H, *J* = 7.50 Hz, CH_3_), 2.72 (t, 2H, *J* = 7.50 Hz, CH_2_), 5.38 (br, 2H, NH_2_), 7.32 (s, 1H, py-H), 7.33 (d, 2H, *J* = 8.40 Hz, C_6_H_2_), 7.97(d, 2H, *J* = 8.40 Hz, C_6_H_2_). IR (KBr), ν (cm^−1^): 3323, 3203, 1640, 1596. MS (EI), *m*/*z*: 267 (M^+^, 100%).

*4-(2,4-Dimethylphenyl)-6-trifluoromethyl-2-aminopyrimidine* (**III-12**): White crystals, yield 72%, m.p. 128–129 °C. ^1^H-NMR (CDCl_3_, 600 MHz), δ (ppm): 2.32 (s, 3H, CH_3_), 2.36 (s, 3H, CH_3_), 7.04 (s, 1H, py-H), 7.11–7.39 (m, 3H, C_6_H_3_), 7.32 (br, 2H, NH_2_). IR (KBr), ν (cm^−1^): 3323, 3208, 1631, 1590. MS (EI), *m*/*z*: 267 (M^+^, 77%).

*4-(4-Fluorophenyl)-6-trifluoromethyl-2-aminopyrimidine* (**III-13**): White crystals, yield 77%, m.p. 168–169 °C (158 °C) (Sareen et al. [22] 1993). ^1^H-NMR (CDCl_3_, 300 MHz), δ (ppm): 5.46 (br, 2H, NH_2_), 7.15–7.22 (m, 2H, C_6_H_2_), 7.29 (s, 1H, py-H), 8.03–8.09 (m, 2H, C_6_H_2_). IR (KBr), ν (cm^−1^): 3323, 3188, 1650, 1591. MS (EI), *m*/*z*: 257 (M^+^, 100%).

*4-(4-Chlorophenyl)-6-trifluoromethyl-2-aminopyrimidine* (**III-14**): White crystals, yield 87%, m.p. 202–203 °C (181–183 °C) (Bonacorso et al. [23], 2008). ^1^H-NMR (CDCl_3_, 300 MHz), δ (ppm): 7.40 (br, 2H, NH_2_), 7.56 (s, 1H, py-H), 7.61 (d, 2H, *J* = 8.40 Hz, C_6_H_2_), 8.21 (d, 2H, *J* = 8.40 Hz, C_6_H_2_). IR (KBr), ν (cm^−1^): 3318, 3186, 1642, 1596. MS (EI), *m*/*z*: 273 (M^+^, 100%).

*4-(4-Bromophenyl)-6-trifluoromethyl-2-aminopyrimidine* (**III-15**): Yellow crystals, yield 63%, m.p. 222–223 °C. ^1^H-NMR (CDCl_3_, 300 MHz), δ (ppm): 3.33 (s, 3H, CH_3_), 7.41 (br, 2H, NH_2_), 7.52 (s, 1H, py-H), 7.75 (d, 2H, *J* = 8.40 Hz, C_6_H_2_), 8.13 (d, 2H, *J* = 8.40Hz, C_6_H_2_); IR (KBr), ν (cm^−1^): 3318, 3203, 1641, 1596. MS (EI), *m*/*z*: 319 (M^+^, 100%).

*4-(4-Trifluoromethylphenyl)-6-trifluoromethyl-2-aminopyrimidine* (**III-16**): White crystals, yield 86%, m.p. 181–182 °C. ^1^H-NMR (CDCl_3_, 300 MHz), δ (ppm): 7.50 (br, 2H, NH_2_), 7.64 (s, 1H, py-H), 7.91 (d, 2H, *J* = 8.40 Hz, C_6_H_2_), 8.38 (d, 2H, *J* = 8.40 Hz, C_6_H_2_); IR (KBr), ν (cm^−1^): 3323, 3213, 1646, 1586. MS (EI), *m*/*z*: 307 (M^+^, 100%).

*4-(3,4-Dichlorophenyl)-6-trifluoromethyl-2-aminopyrimidine* (**III-17**): White crystals, yield 69%, m.p. 197–198 °C. ^1^H-NMR (CDCl_3_, 300 MHz), δ (ppm): 7.47 (br, 2H, NH_2_), 7.67 (s, 1H, py-H), 7.81 (d, 1H, *J* = 8.40 Hz, C_6_H), 8.19 (dd, 1H, *J* = 8.40, 2.10 Hz, C_6_H), 8.46 (d, 1H, *J* = 2.10Hz, C_6_H). IR (KBr), ν (cm^−1^): 3323, 3208, 1646, 1586. MS (EI), *m*/*z*: 307 (M^+^, 100%).

*4-(3-Fluoro-4-methoxyphenyl)-6-trifluoromethyl-2-aminopyrimidine* (**III-18**): White crystals, yield 64%, m.p. 175–176 °C. ^1^H-NMR (CDCl_3_, 300 MHz), δ (ppm): 3.96 (s, 3H, OCH_3_), 5.42 (br, 2H, NH_2_), 7.04–7.88 (m, 4H, C_6_H_3_ + py-H). IR (KBr), ν (cm^−1^): 3333, 3213, 1631, 1596. MS (EI), *m*/*z*: 287 (M^+^, 100%).

*4-(3,4-Difluoro-5-methoxyphenyl)-6-trifluoromethyl-2-aminopyrimidine* (**III-19**): White crystals, yield 73%, m.p. 198–199 °C. ^1^H-NMR (CDCl_3_, 300 MHz), δ (ppm): 4.10 (s, 3H, OCH_3_), 5.45 (br, 2H, NH_2_), 7.22 (s, 1H, py-H), 7.64 (d, 2H, *J* = 9.90 Hz, C_6_H_2_); IR (KBr), ν (cm^−1^): 3328, 3213, 1641, 1591. MS (EI), *m*/*z*: 305 (M^+^, 100%).

*4-(3-Trifluoromethyl-4-methoxyphenyl)-6-trifluoromethyl-2-aminopyrimidine* (**III-20**): White crystals, yield 78%, m.p. 168–169 °C. ^1^H-NMR (CDCl_3_, 300 MHz), δ (ppm): 3.99 (s, 3H, OCH_3_), 7.38 (s, 2H, NH_2_), 7.42–8.51 (m, 4H, C_6_H_3_ + py-H). IR (KBr), ν (cm^−1^): 3318, 3213, 1641, 1596. MS (EI), *m*/*z*: 337 (M^+^, 100%).

*4-(3,5-Bis(trifluoromethyl)phenyl)-6-trifluoromethyl-2-aminopyrimidine* (**III-21**): White crystals, yield 83%, m.p. 142–143 °C. ^1^H-NMR (CDCl_3_, 300 MHz), δ (ppm): 5.49 (br, 2H, NH_2_), 7.38 (s, 1H, py-H), 8.03 (s, 1H, C_6_H_1_), 8.50 (s, 2H, C_6_H_2_); IR (KBr), ν (cm^−1^): 3326, 3217, 1644, 1591. MS (EI), *m*/*z*: 375 (M^+^, 100%).

*4-(3,4-Difluorophenyl)-6-trifluoromethyl-2-aminopyrimidine* (**III-22**): White crystals, yield 65%, m.p. 138–140 °C. ^1^H-NMR (CDCl_3_, 600 MHz), δ (ppm): 5.41 (br, 2H, NH_2_), 7.27 (s, 1H, py-H), 7.26–7.31 (m, 1H, C_6_H_1_), 7.78–7.80 (m, 1H, C_6_H_1_), 7.93–7.97 (m, 1H, C_6_H_1_). IR (KBr), ν (cm^−1^): 3323, 3209, 1645, 1591. MS (EI), *m*/*z*: 275 (M^+^, 100%).

### 3.3. Bioassay of Fungicidal Activity

Fungicidal activity of the title compounds was evaluated using the method given in references [30,31,32].

#### 3.3.1. In Vitro Fungicidal Activity of Compounds **III-1**–**III-22** against *B. cinerea* (The Mycelium Growth Test)

The *B. cinerea* strain was isolated from damaged tomato parts from a greenhouse in Shenyang, Liaoning Province, China, in April 2013, and cultured on potato dextrose agar (PDA) for many generations. The culture media were fructose gelatin agar (FGA) [33] and potato dextrose agar (PDA). The final concentrations of compounds were 50, 12.5, 3.13, 0.78 μg/mL on FGA, and 100, 25, 6.25, 1.56, 0.39 μg/mL on PDA. The commercial reference fungicides were pyrimethanil and cyprodinil. The EC_50_ and EC_80_ values of compounds **III-1** to **III-22** were estimated using logit analysis, and the results given in Table 1.

#### 3.3.2. In Vivo Fungicidal Activity of Compounds **III-1**–**III-22** against *B. cinerea* (Mycelium Inoculation Method)

In vivo, the concentration of compounds was 750 μg/mL. The results of compounds **III-1** to **III-22** against *B. cinerea* by pot culture test method (mycelium inoculation method) [32,34] in greenhouse were shown in Table 2.

### 3.4. Mode of Action

High activity compound **III-3** was screen out by the above method and chosen for mode of action studies.

#### 3.4.1. Toxicity Test of Compound **III-3** Against Five Pyrimethanil-resistant Strains of *B.* *cinerea*

CY-12, HLD-18, HLD-15, FS-09 and DL-11were five pyrimethanil-resistant strains of *B.* *cinerea*, which were respectively isolated from damaged tomato parts from a greenhouse in Chaoyang, Huludao, Fushun and Dalian, Liaoning Province, China, in April 2014. The effect of compound **III-3** against pyrimethanil-resistant strains of *B. cinerea* was assessed on FGA, and the results are shown in Table 3.

#### 3.4.2. Spore Yield of *B. cinerea* Test Method

The concentration gradient was 80, 20, 5 μg/mL. The plugs of *B. cinerea* were inoculated on PDA, and they were cultured for ten days at 23 °C. After a large number of spores were produced in the blank control, the conidial suspensions were prepared by seeding conidia in a 0.05% Tween 80 solution with 2 mL sterile water. Spores were determined directly on the hemocytometer. Spore production was calculated according to Equation (1). The spore yield inhibition rate was calculated according to Equation (2):

(1) $$Q=Q_{0}\times 10^{6}/mL$$

(2) $$I=(1-\frac{G_{1}}{G_{0}})\times 100\%$$

where *Q* is spore production quantity in per milliliter conidial suspension, *Q*_0_ is the average number of spores each small lattice. *I* is the spore yield inhibition rate, *G*_0_ is the average spore production in the blank test, *G*_1_ is the average spore production in the presence of compounds.

#### 3.4.3. The Spore Germination Test Method

The effect of compound **III-3** on spore germination against *B. cinerea* was determined in concave slides by liquid drop method. The method was given in reference [32]. Concentration gradient was 100, 50, 25, 12.5, 6.25 μg/mL. The commercial fungicide pyrimethanil was used as the positive control, and the results were given in Figure 5.

#### 3.4.4. Effect of Compound **III-3** on the Sclerotia Production of *B. cinerea*

Concentration gradient was 40, 20, 10, 5 μg/mL. The commercial fungicide cyprodinil was used as the positive control, and the results are given in Figure 6.

#### 3.4.5. In Vivo Protective and Therapeutic Effects of Compound **III-3** Against *B. cinerea* (Spore Inoculation method)

The spore inoculation method was given in reference [29]. Preventive and therapeutic effects of the compound **III-3** were tested against *B.* *cinerea* in cucumber (200 μg/mL) and tomato (100 μg/mL). The results are shown in Figure 7.

#### 3.4.6. Preparation of Scanning Electron Microscopy Samples

The method was given in reference [35,36]. Spore suspensions (1 mL, 5 × 10^5^ spores/mL) were inoculated for PD cultivation on medium which contained compound **III-3**. Final concentrations were 0.78, 3.125, 12.5 μg/mL. The commercial fungicide cyprodinil was used as positive control. The samples were shook culture at 23 °C, 140 r/min. Then the mycelia were randomly selected after 72 h. The samples were fixed in 3%–4% glutaraldehyde and washed with phosphate buffer solution (PBS pH 6.8) 4–6 times for each time at intervals of 20–30 min to remove the debris. Then the samples were dehydrated with acetone series (30%, 50%, 70%, 80%, 90%, 95% and 100%) at intervals of 30 min, which were dehydrated 100% acetone three times. Finally, the samples were treated by CO_2_ critical point drying, sticky sample, and sputter coated with a thin layer of gold. The ultrastructure of samples was observed using a BCPCAS4800 scanning electronic microscope (Hitachi, Tokyo, Japan).

#### 3.4.7. Preparation of Transmission Electron Microscopy Samples

Culture and pretreatment of samples were the same as the method of Section 3.4.6. Final concentrations of compound **III-3** and cyprodinil were 20, 40 μg/mL. The mycelia were randomly selected after 120 h. The uttrastructure of samples was observed using a Hitachi transmission electron microscope (Hitachi, Tokyo, Japan).

## 4. Conclusions

In summary, based on our previous structure-activity relationship studies, a series of 4-phenyl-6-trifluoromethyl-2-aminopyrimidines were designed and synthesized. The title compounds exhibited high in vitro and in vivo fungicidal activity against *B.* *cinerea*, not only against the sensitive strain but also against resistant strains. Compound **III-3** had strong inhibition effect on the mycelium growth, spore production, spore germination and sclerotia production of *B.* *cinerea*, which indicated excellent prevention and treatment effects on gray mold. The preliminary structure-activity relationships demonstrated that the substituents at the *ortho*-position of phenyl ring favored the bioactivity, especially a fluorine atom. The effects of compound **III-3** on the mycelial morphology and ultrastructure of *B.* *cinerea* were different from that of cyprodinil. Further research is necessary to understand its site of action.

## Figures and Tables

**Table 1:** The fungicidal activity of compounds **III-1**–**III-22** against *B. cinerea.*

Compound	R^2^	FGA		PDA	
		EC_50_/(μg/mL)	EC_80_/(μg/mL)	EC_50_/(μg/mL)	EC_80_/(μg/mL)
**III-1**	H	48.1	>100	223.5	>10^3^
**III-2**	2-CH_3_	5.3	18.1	20.3	125.5
**III-3**	2-F	0.8	5.1	0.6	3.9
**III-4**	2-Cl	12.1	36.9	3.3	35.4
**III-5**	2-Br	26.1	48.8	17.8	71.5
**III-6**	2-CF_3_	49.0	133.1	95.1	>10^3^
**III-7**	2-OCH_3_	27.2	99.0	9.4	88.8
**III-8**	2-OH	42.3	100.1	403.9	>10^3^
**III-9**	4-OCH_3_	>100	>100	348.4	>10^3^
**III-10**	4-CH_3_	18.0	72.1	846.9	>10^3^
**III-11**	4-CH_2_CH_3_	2.6	14.8	202.2	>10^3^
**III-12**	2,4-(CH_3_)_2_	3.1	14.6	16.3	>10^3^
**III-13**	4-F	1.3	6.3	135.6	>10^3^
**III-14**	4-Cl	55.5	>100	497.1	>10^3^
**III-15**	4-Br	4.6	38.8	155.6	>10^3^
**III-16**	4-CF_3_	0.3	19.6	>10^3^	>10^3^
**III-17**	3,4-Cl_2_	/	/	46.5	555.3
**III-18**	3-F, 4-OCH_3_	3.3	>100	69.4	>10^3^
**III-19**	3,4-F_2_, 5-OCH_3_	>100	>100	3.4	277.8
**III-20**	3-CF_3_, 4-OCH_3_	17.2	>100	42.9	441.4
**III-21**	3,5-(CF_3_)_2_	1.1	50.4	808.7	>10^3^
**III-22**	3, 4-F_2_	0.3	18.5	120.5	>10^3^
cyprodinil		32.9	177.8	17.8	175.8
pyrimethanil		24.0	76.2	7.5	180.1

**Table 2:** Control efficiency of compounds **III-1**–**III-22** against *B*. *cinerea* at 750 μg/mL (mycelium inoculation method).

Compound	R^2^	Control Efficiency on Leaves of Cucumber (%) (±SEM)
**III-1**	H	n
**III-2**	2-CH_3_	n
**III-3**	2-F	98 ± 2 a
**III-4**	2-Cl	73 ± 6 c
**III-5**	2-Br	79 ± 5 bc
**III-6**	2-CF_3_	59 ± 8 c
**III-7**	2-OCH_3_	71 ± 7 c
**III-8**	2-OH	95 ± 3 ab
**III-9**	4-OCH_3_	52 ± 18 bcde
**III-10**	4-CH_3_	69 ± 8 ab
**III-11**	4-CH_2_CH_3_	79 ± 4 ab
**III-12**	2,4-(CH_3_)_2_	44 ± 19 de
**III-13**	4-F	68 ± 6 ab
**III-14**	4-Cl	n
**III-15**	4-Br	20 ± 28 e
**III-16**	4-CF_3_	45 ± 18 de
**III-17**	3,4-Cl_2_	n
**III-18**	3-F, 4-OCH_3_	24 ± 25 e
**III-19**	3,4-F_2_, 5-OCH_3_	n
**III-20**	3-CF_3_, 4-OCH_3_	25 ± 9 e
**III-21**	3,5-(CF_3_)_2_	n
**III-22**	3,4-F_2_	52 ± 14 bcde
cyprodinil		64 ± 9 ab
pyrimethanil		47 ± 10 cde

The letters a–e denoted the results of difference significance analysis. Means followed by the same letter within the same column are not significantly different (*p* > 0*.*05, Fisher′s LSD multiple comparison test). n: no activity.

**Table 3:** The fungicidal activity of compound **III-3** against resistent *strains of B. cinerea* (FGA).

Resistent Strains of *B.* *cinerea*	Compound	EC_50_ (μg/mL)	Relative Toxicity (EC_50_)	EC_80_ (μg/mL)	Relative Toxicity (EC_80_)
CY-12	**III-3**	1.0	35.8	32.2	2.9
	cyprodinil	9.7	3.7	60.9	1.5
	pyrimethanil	36.1	1.0	92.2	1.0
HLD-18	**III-3**	3.8	11.7	20.6	7.1
	cyprodinil	25.4	1.7	56.8	2.6
	pyrimethanil	44.4	1.0	145.4	1.0
HLD-15	**III-3**	1.42	11.3	10.96	12.1
	cyprodinil	34.05	0.5	87.29	1.52
	pyrimethanil	16.11	1	132.64	1
FS-09	**III-3**	1.19	8.1	22.29	51.4
	cyprodinil	7.59	1.3	49.79	23.0
	pyrimethanil	9.67	1	1146.44	1
DL-11	**III-3**	2.74	8.5	23.72	8.0
	cyprodinil	8.06	2.9	54.9	3.5
	pyrimethanil	23.37	1	190.77	1

## Supplementary Materials

Supplementary materials can be accessed at: http://www.mdpi.com/1420-3049/21/7/828/s1.
