# Supplementary material for: Synthesis, Fungicidal Activity and Mode of Action of 4-Phenyl-6-trifluoromethyl-2-aminopyrimidines against Botrytis cinerea
Source: Molecules. 2016 Jun 24;21(7):828. doi: 10.3390/molecules21070828 (PMC6273116; doi:10.3390/molecules21070828)
Supplement: Supplementary file 1 [file molecules-21-00828-s001.pdf]

Supplementary Materials: Synthesis, Fungicidal Activity and Mode of Action of4-Phenyl-6-trifluoromethyl-2-aminopyrimidines Against *Botrytis cinerea*

Chunhui Liu, Zining Cui, Xiaojing Yan, Zhiqiu Qi, Mingshan Ji and Xinghai Li

^{1}H-NMR spectra of compounds III-1–III-22

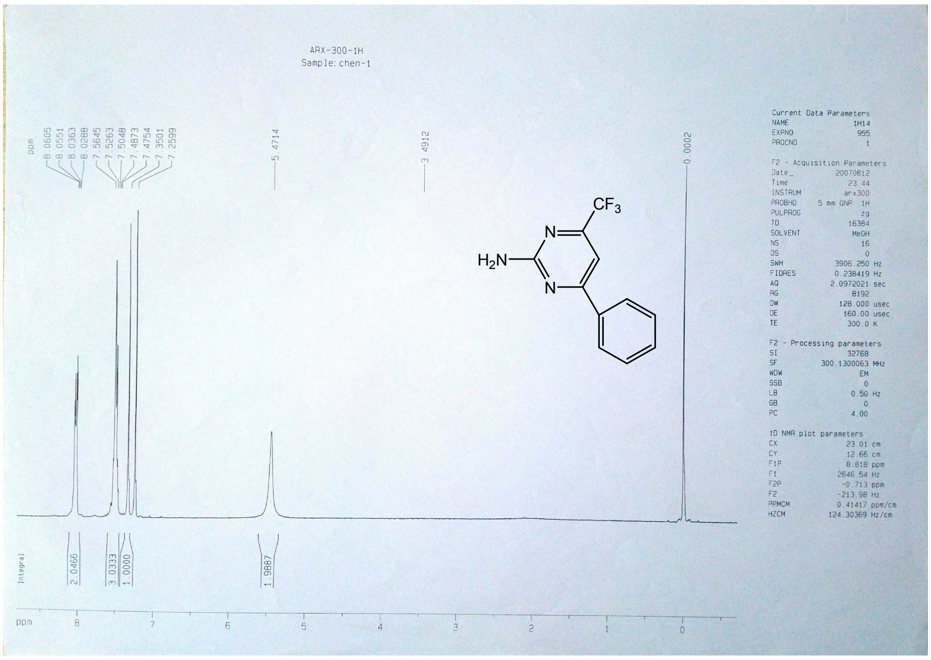

Figure S1. ^{1}H-NMR spectrum of compound **III-1**.

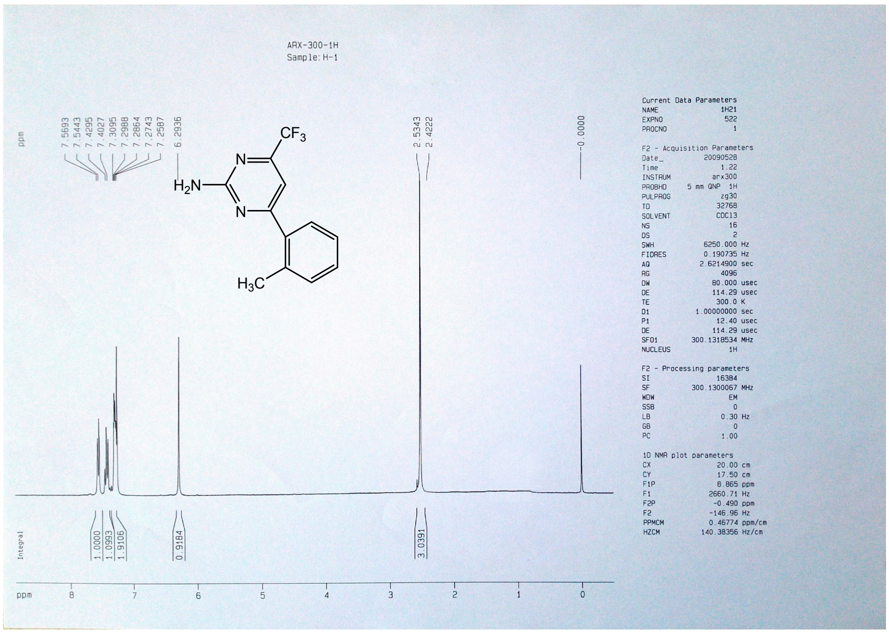

Figure S2. ^{1}H-NMR spectrum of compound **III-2**.

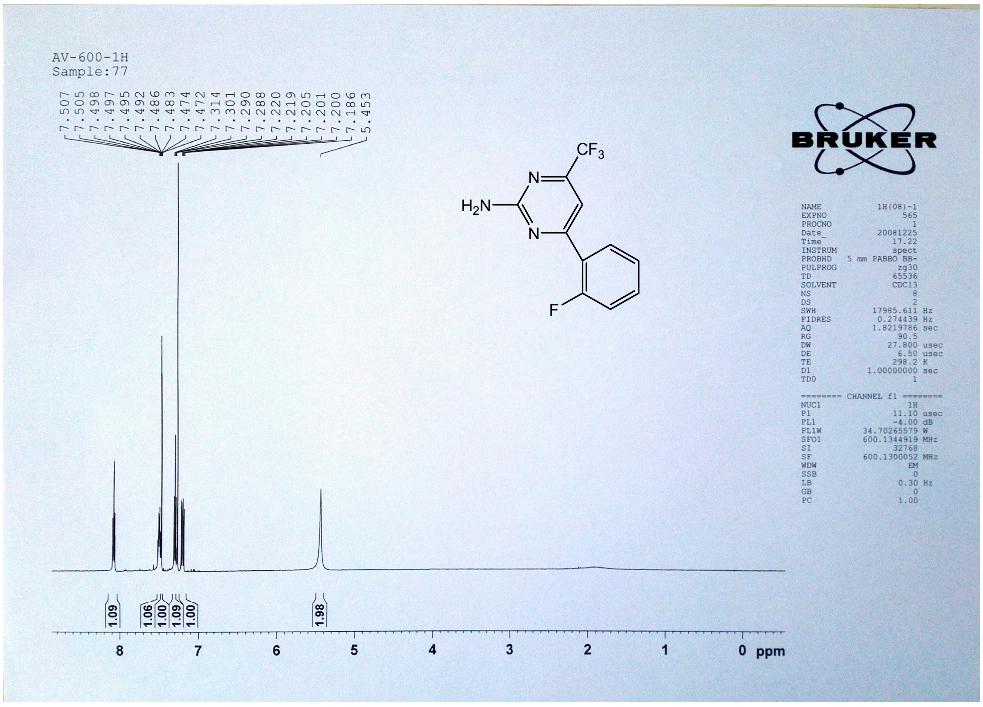

Figure S3. ^{1}H-NMR spectrum of compound **III-3**.

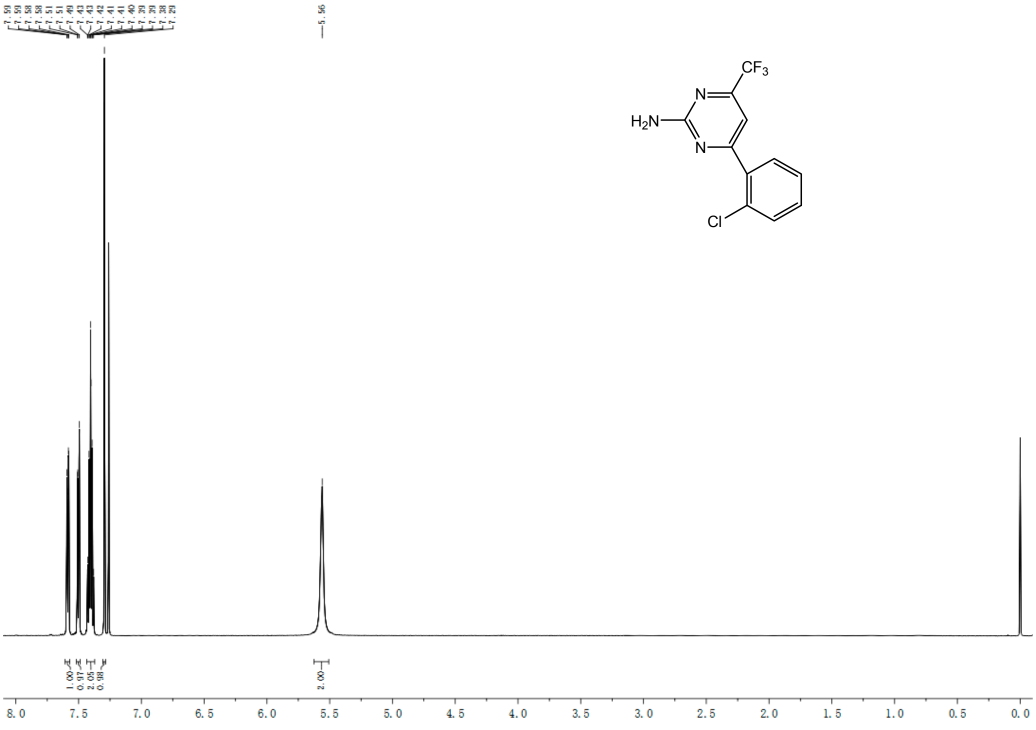

Figure S4. ^{1}H-NMR spectrum of compound **III-4**.

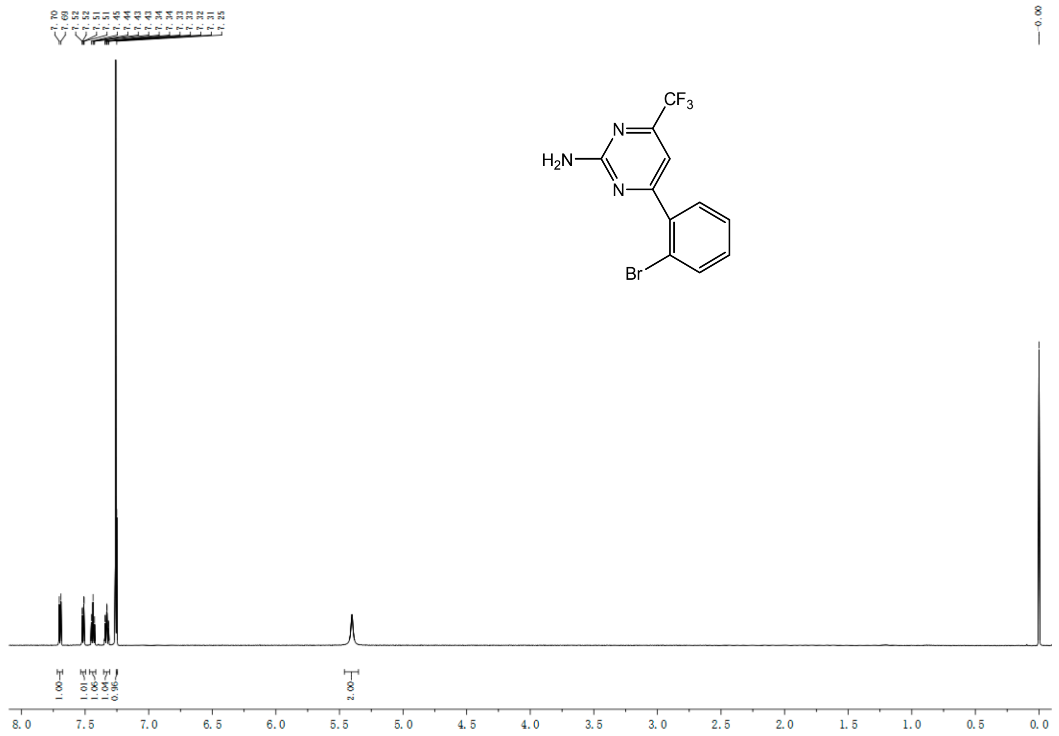

Figure S5. ^{1}H-NMR spectrum of compound **III-5**.

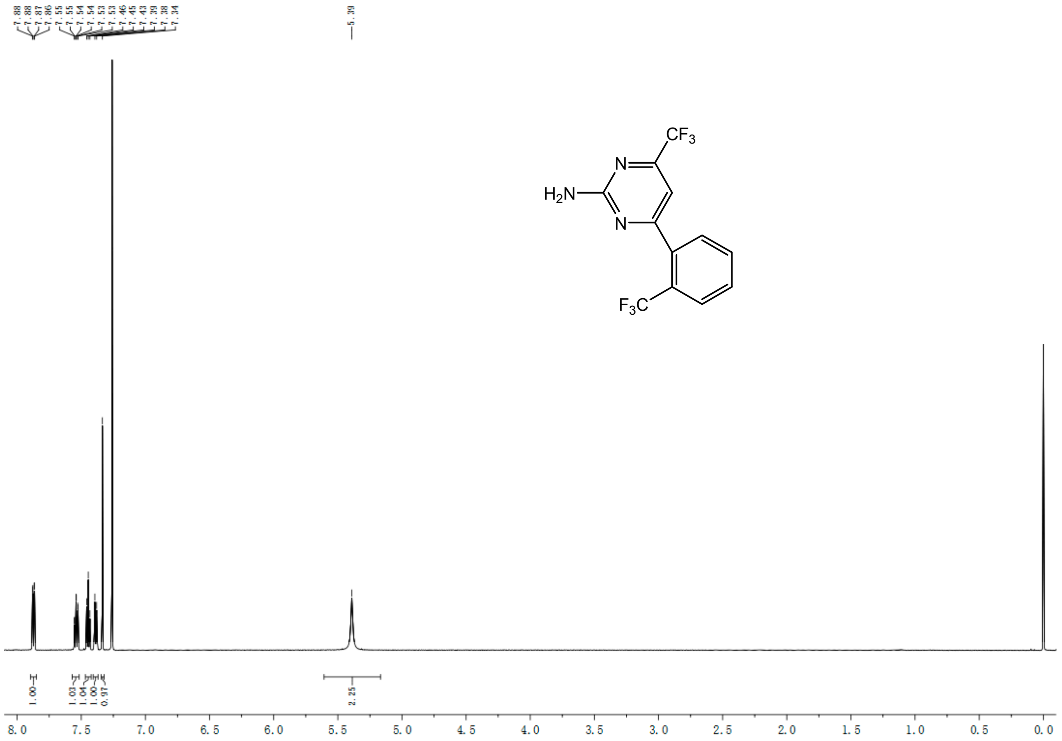

Figure S6. ^{1}H-NMR spectrum of compound **III-6**.

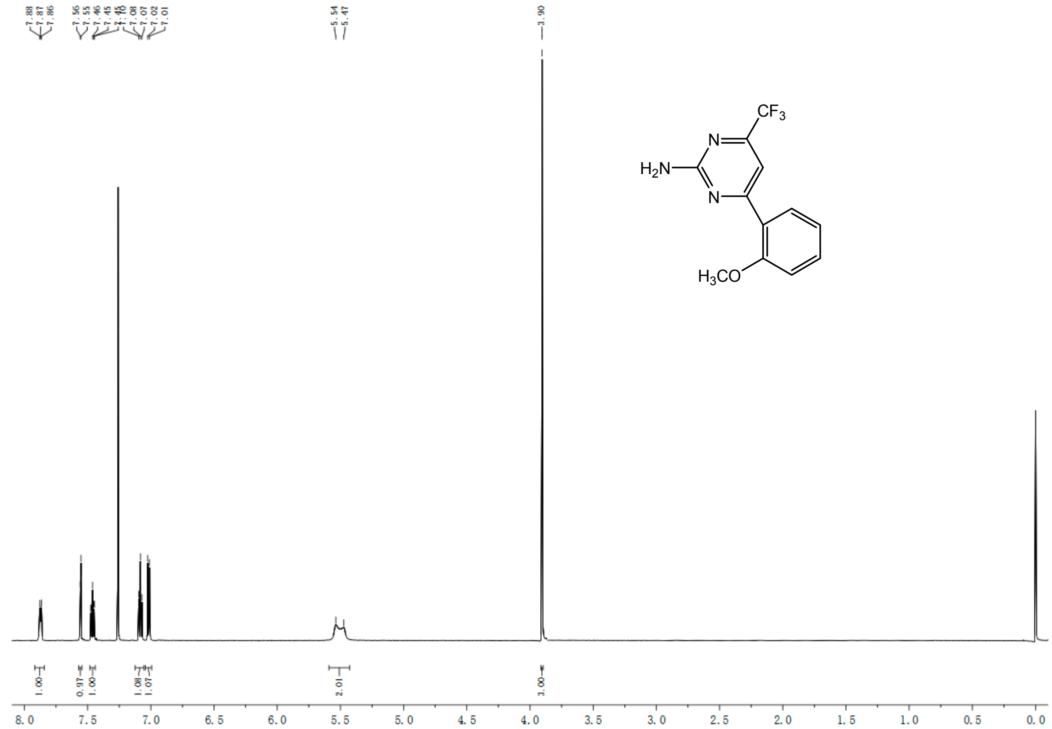

Figure S7. ^{1}H-NMR spectrum of compound **III-7**.

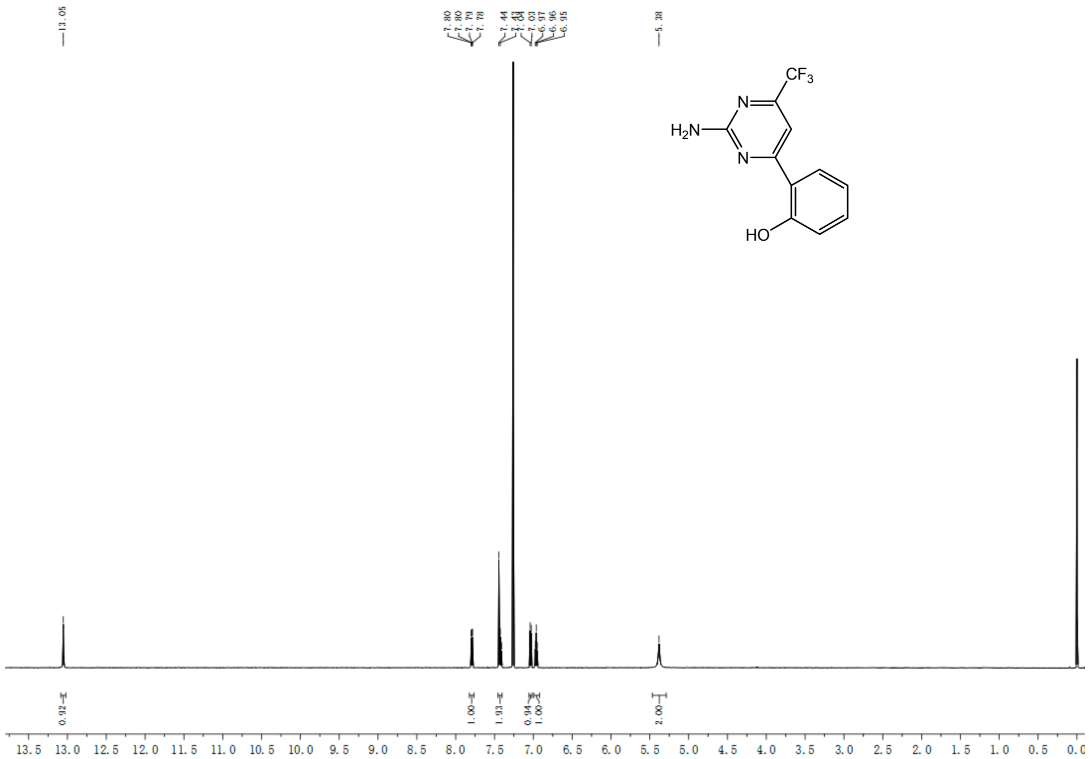

Figure S8. ^{1}H-NMR spectrum of compound **III-8.**

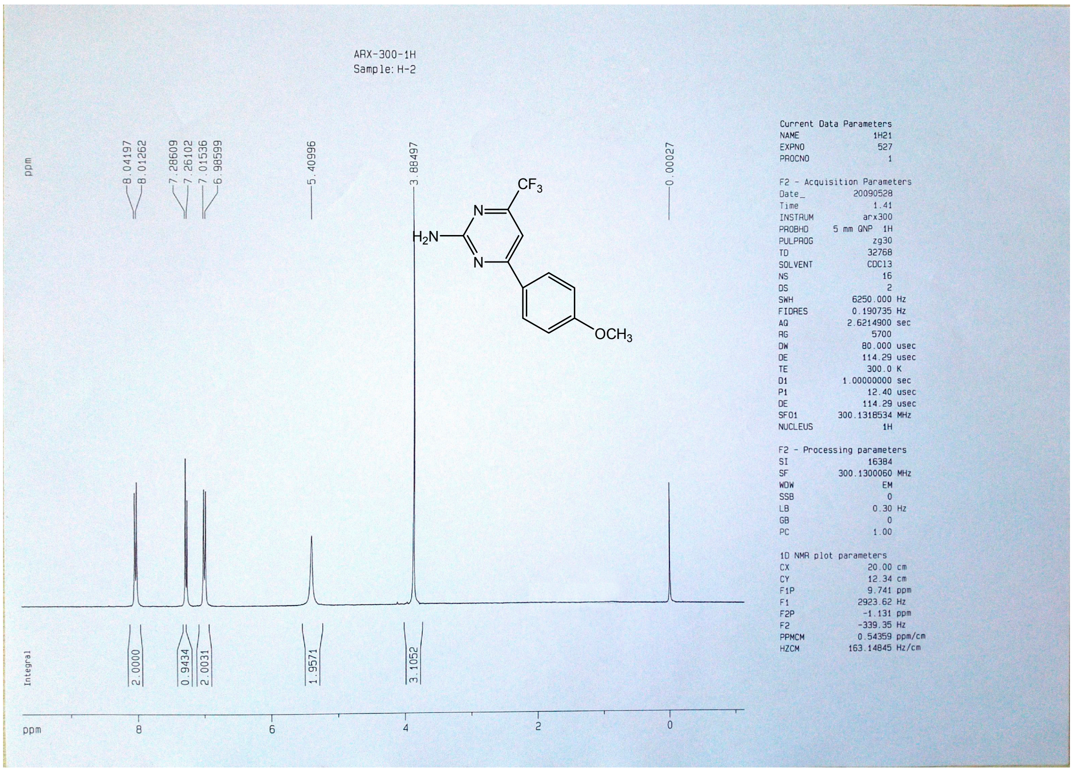

Figure S9. ^{1}H-NMR spectrum of compound **III-9**.

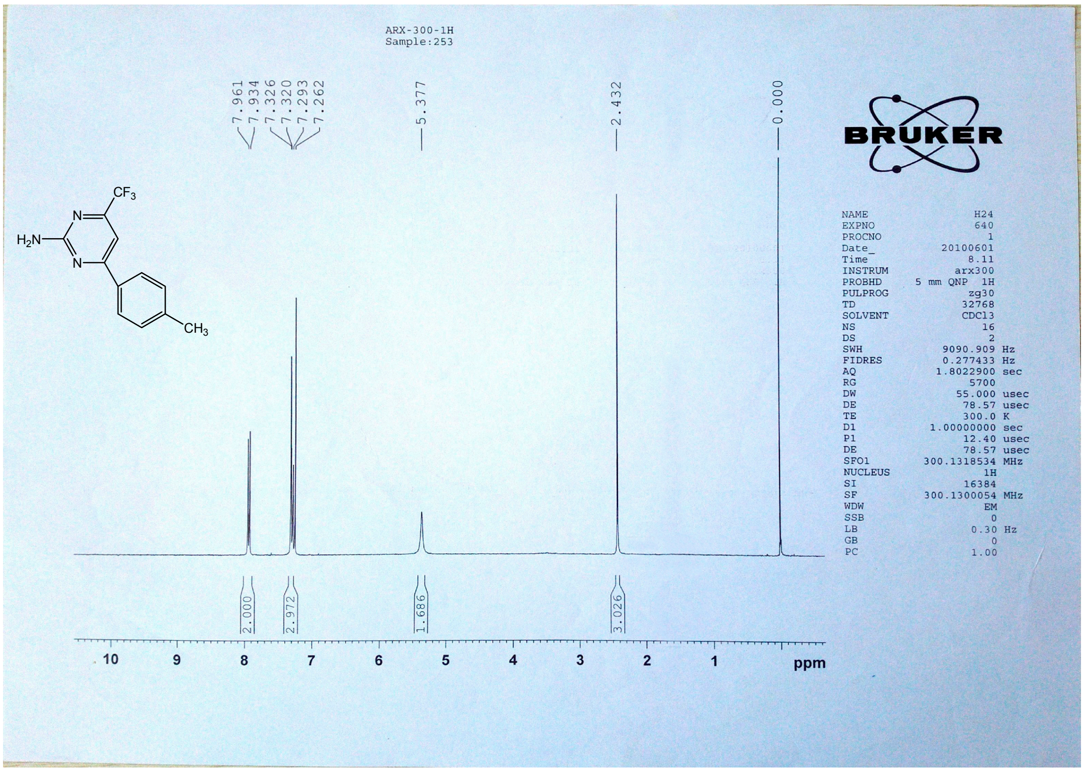

Figure S10. ^{1}H-NMR spectrum of compound **III-10**.

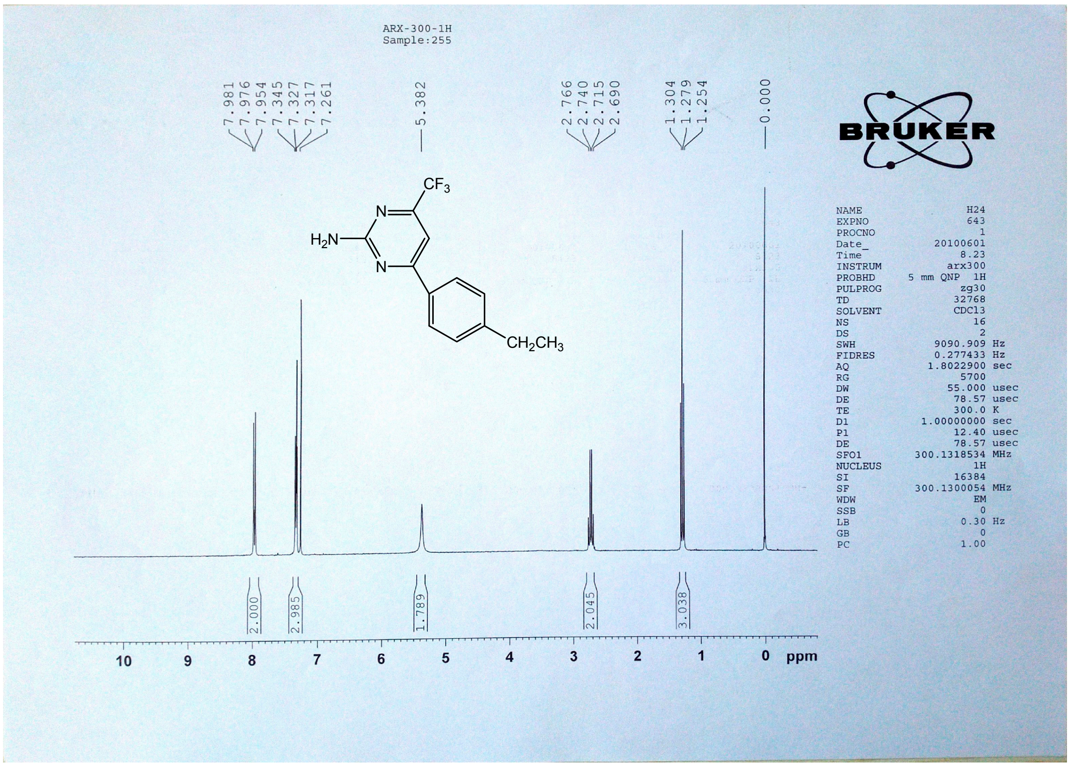

Figure S11. ^{1}H-NMR spectrum of compound **III-11**.

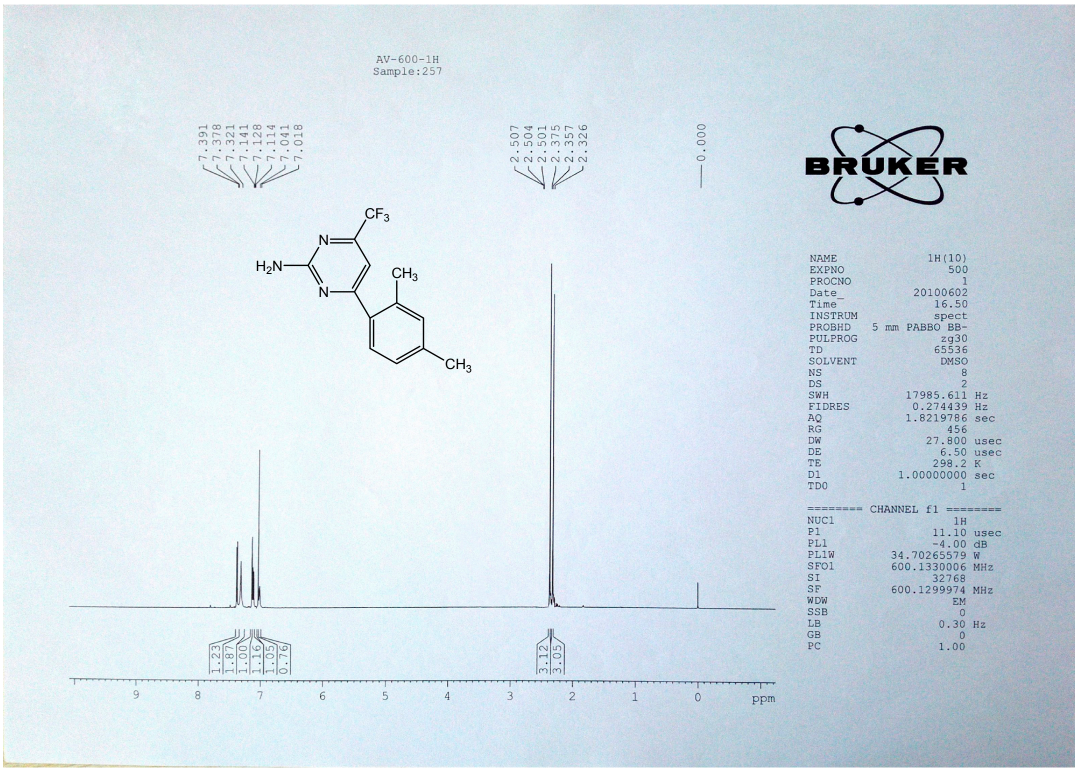

Figure S12. ^{1}H-NMR spectrum of compound **III-12**.

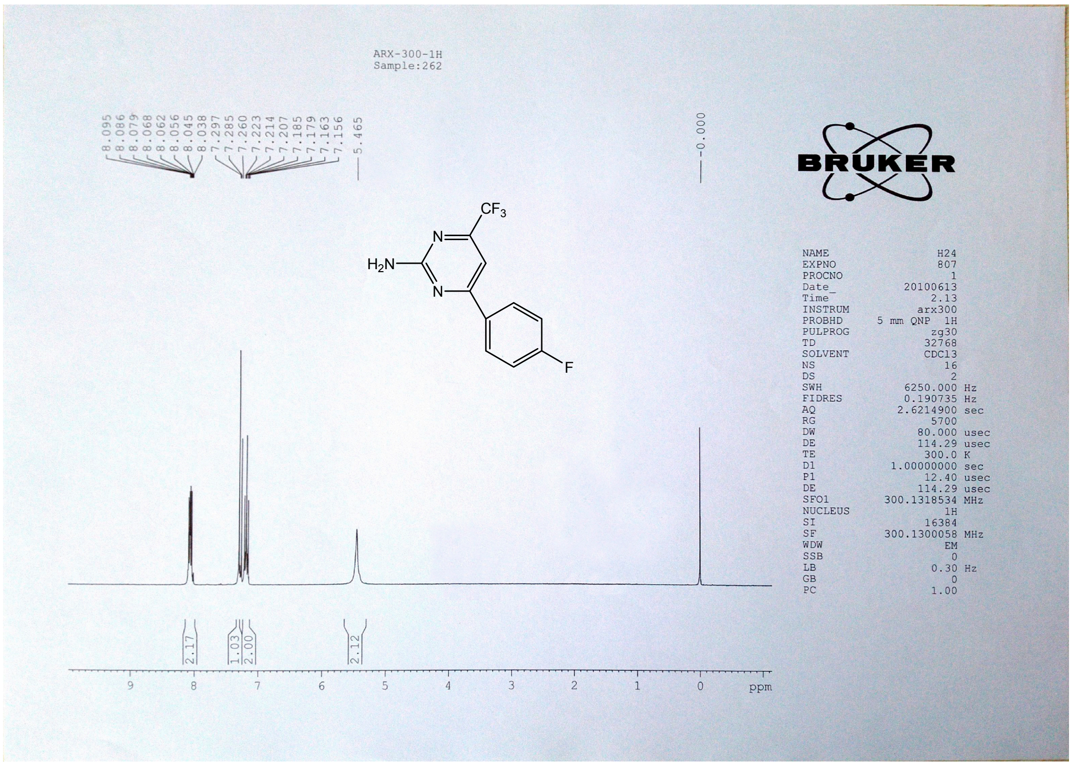

Figure S13. ^{1}H-NMR spectrum of compound **III-13**.

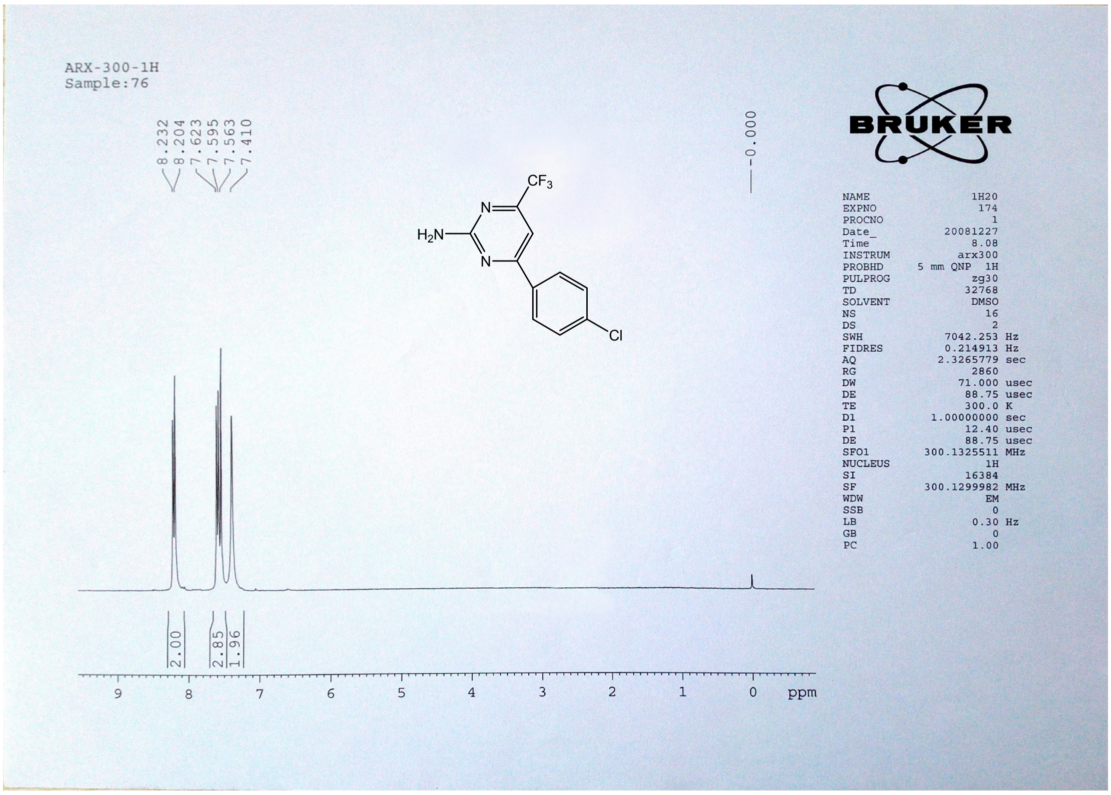

Figure S14. ^{1}H-NMR spectrum of compound **III-14**.

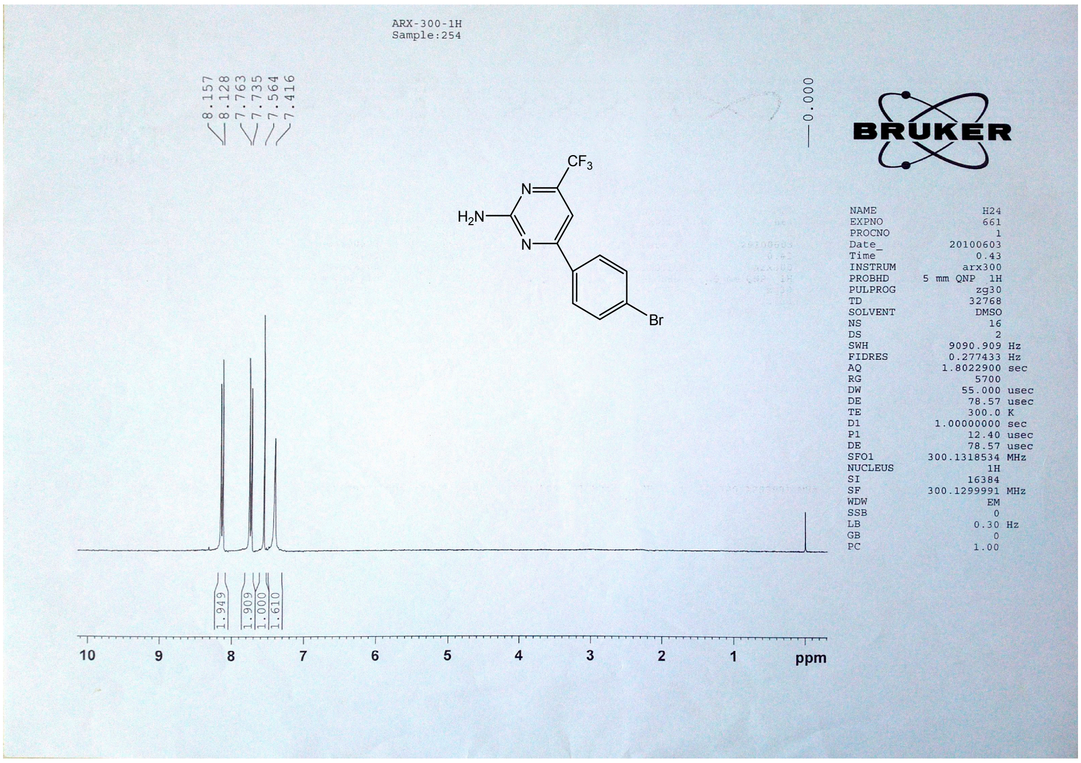

Figure S15. ^{1}H-NMR spectrum of compound **III-15**.

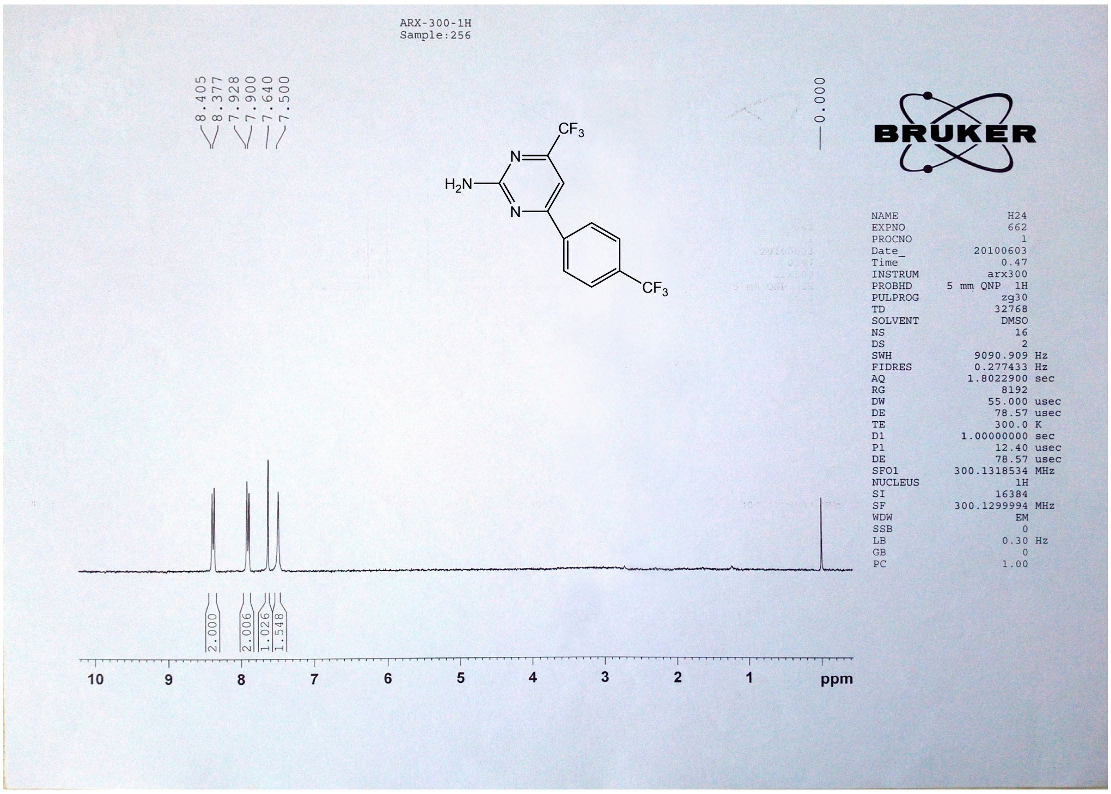

Figure S16. ^{1}H-NMR spectrum of compound **III-16**.

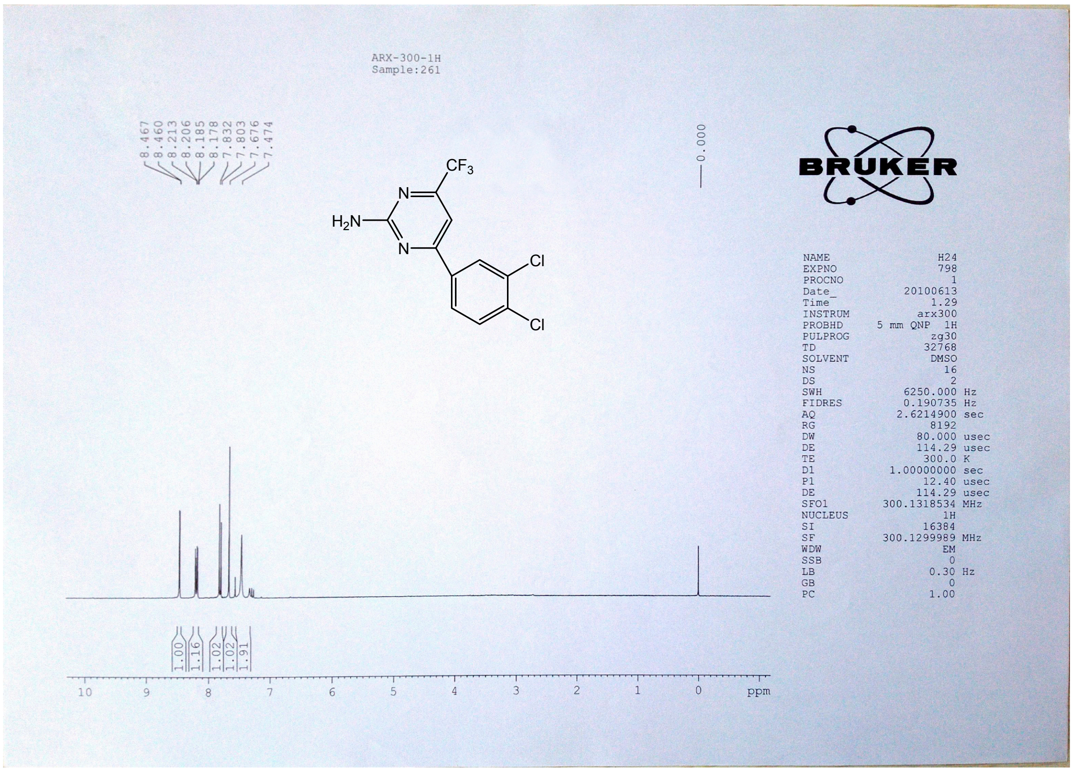

Figure S17. ^{1}H-NMR spectrum of compound **III-17**.

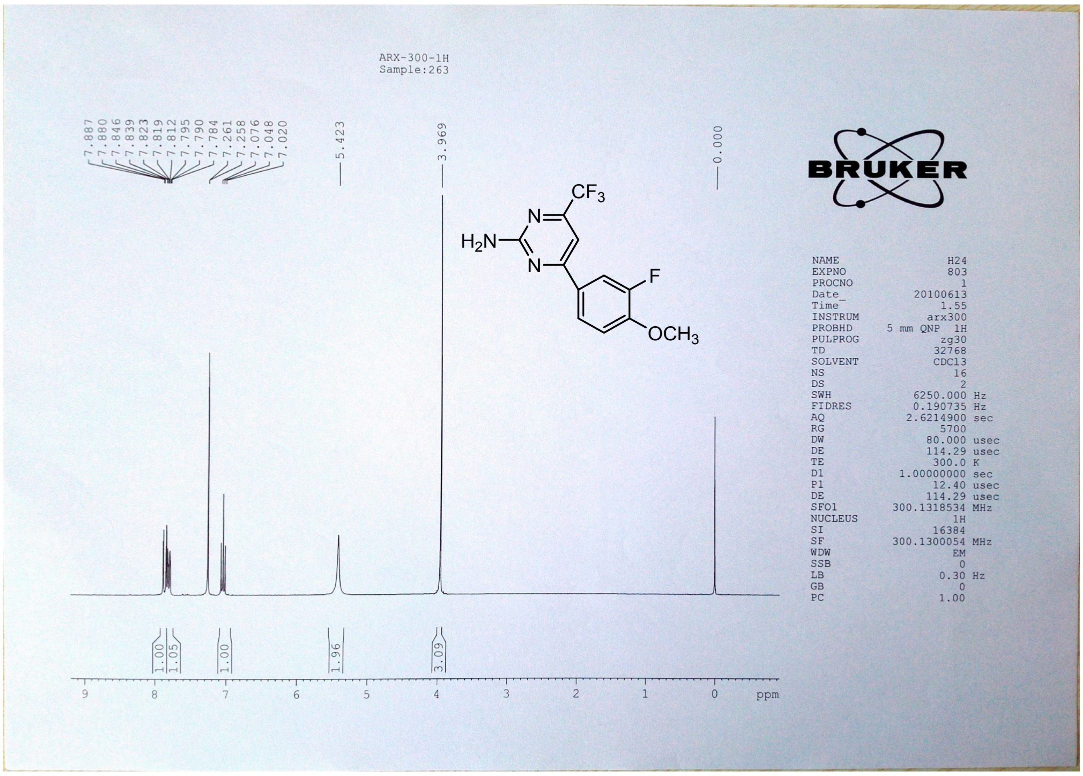

Figure S18. ^{1}H-NMR spectrum of compound **III-18**.

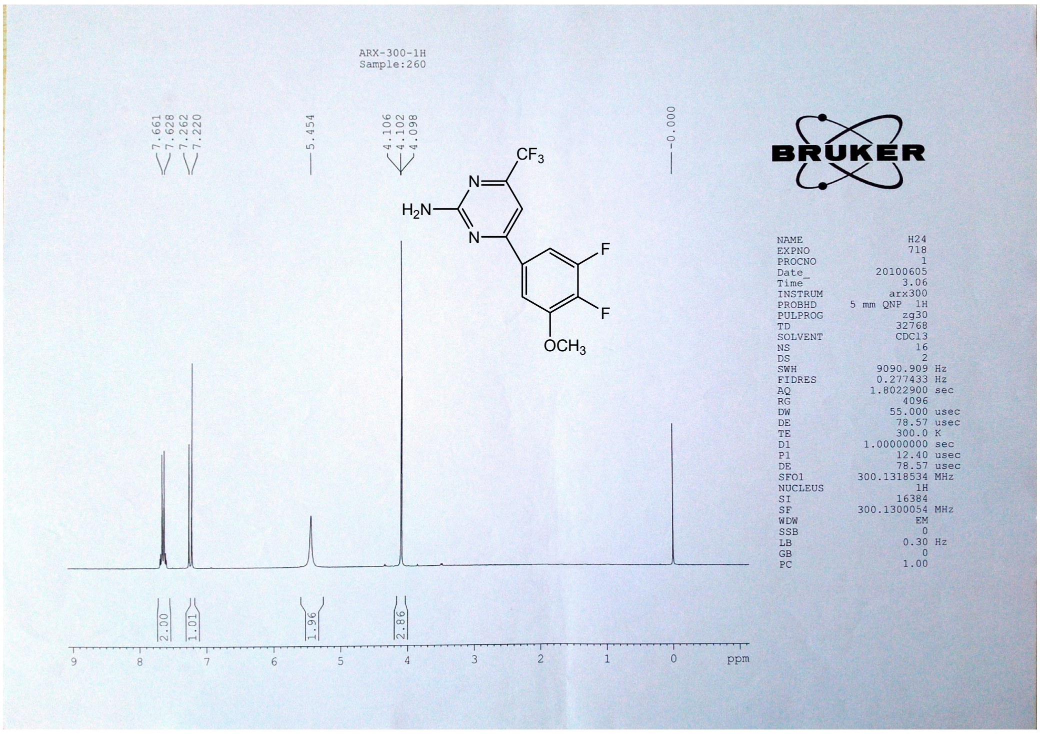

Figure S19. ^{1}H-NMR spectrum of compound **III-19**.

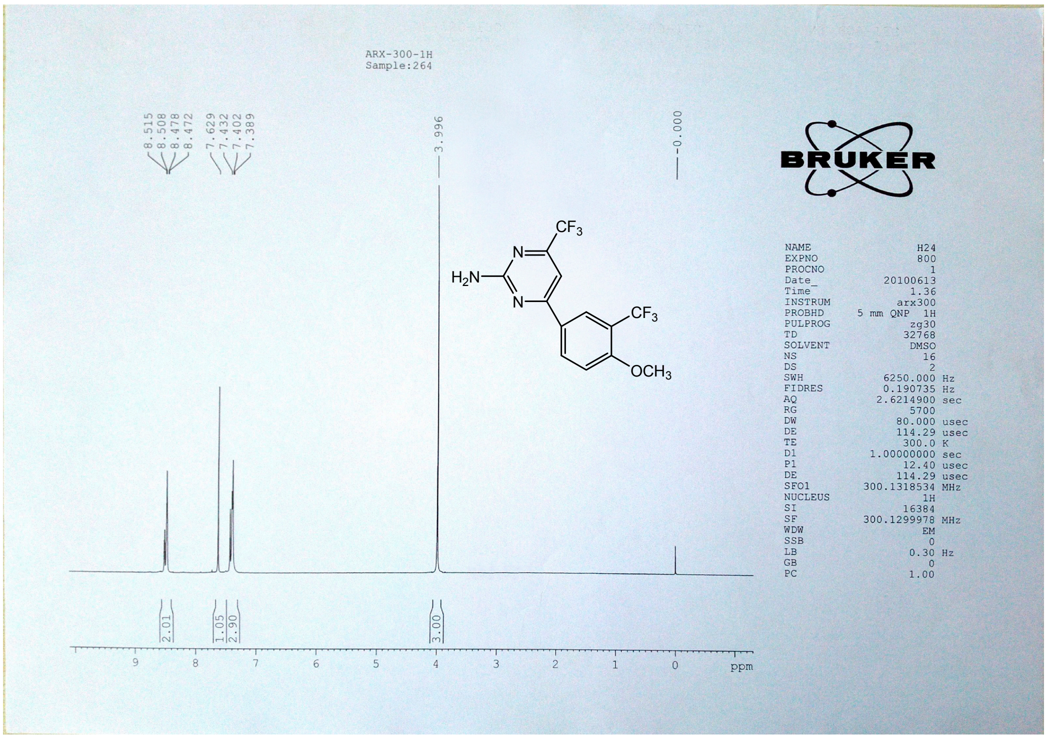

Figure S20. ^{1}H-NMR spectrum of compound **III-20**.

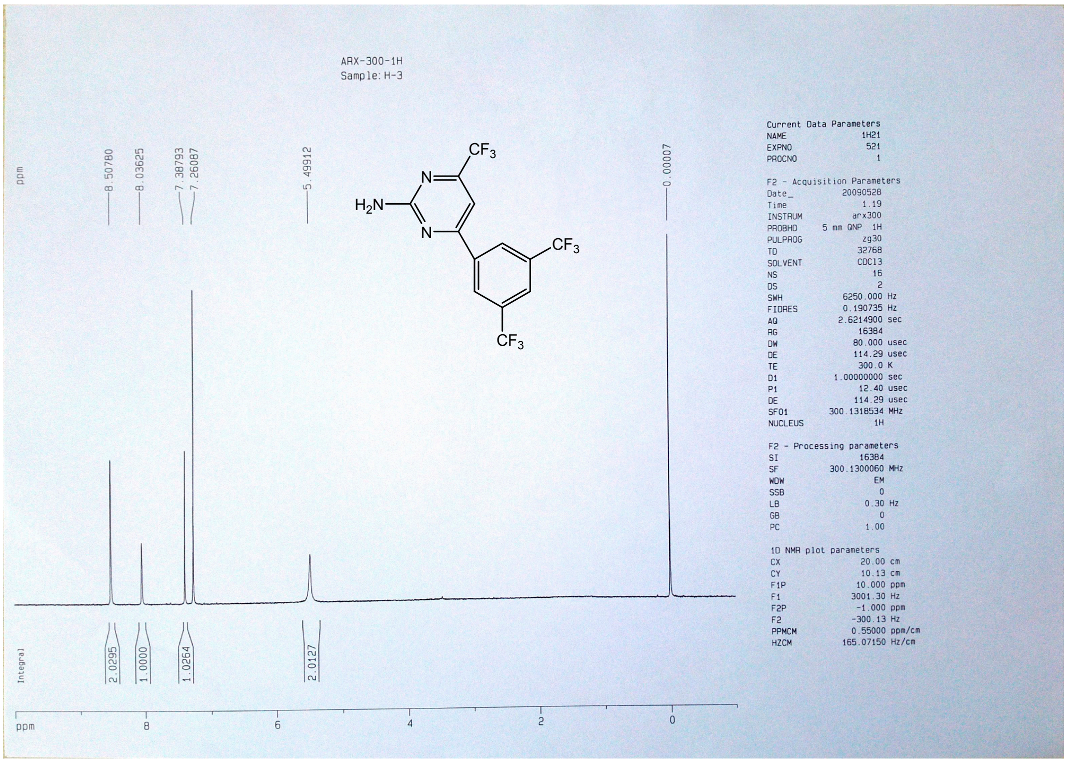

Figure S21. ^{1}H-NMR spectrum of compound **III-21**.

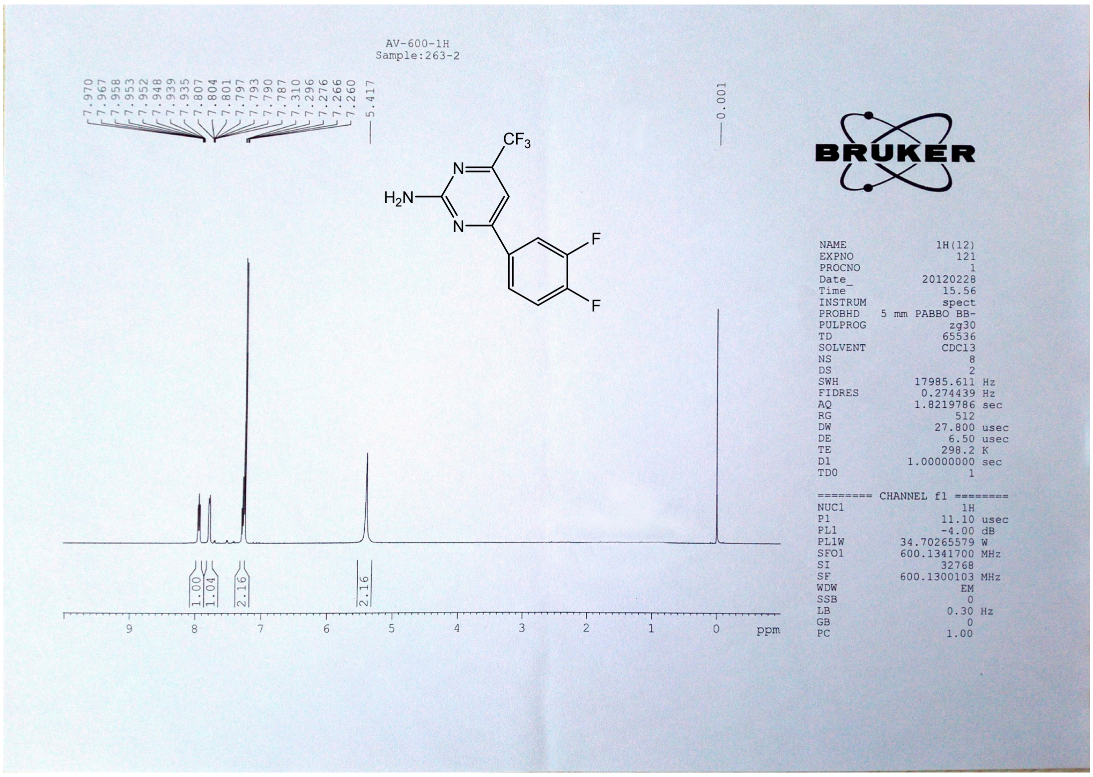

Figure S22. ^{1}H-NMR spectrum of compound **III-22**.
